# Oral preexposure prophylaxis use and the risk of bacterial sexually transmitted infections and HIV among African women: A prospective observational cohort study

**DOI:** 10.1371/journal.pmed.1004962

**Published:** 2026-03-09

**Authors:** David Mukasa, John Kinuthia, Allison Meisner, Daniel Matemo, Torin Schaafsma, Jennifer Morton, Cynthia Wandera, Elvira Budiawan, Valarie Kemuto, Cherotich Irine, Stephen Odhiambo, Mercy Bii, Beatrice Oduor, Esther Achieng, Tessy Oyombra, Ugochinyere Vivian Ukah, Kenneth K. Mugwanya, for the FP Plus Project Team

**Affiliations:** 1 Department of Global Health, University of Washington, Seattle, United States of America; 2 Research & Programs, Kenyatta National Hospital, Nairobi, Kenya; 3 Vaccine and Infectious Diseases Division, Fred Hutchinson Cancer Center, Seattle, United States of America; 4 Department of Medicine, McGill University, Montréal, Quebec, Canada; 5 Department of Epidemiology, University of Washington, Seattle, United States of America; Boston University School of Public Health, UNITED STATES OF AMERICA

## Abstract

**Background:**

Oral preexposure prophylaxis (PrEP) effectively reduces HIV incidence when used with sufficient adherence, but does not protect against bacterial sexually transmitted infections (STIs). Several studies have documented high rates of bacterial STIs among individuals initiating and using PrEP. We evaluated the association between PrEP use and the risk of STI among African women accessing family planning clinics.

**Methods and findings:**

We conducted a prospective cohort study nested within a large pragmatic stepped-wedge cluster randomized trial of PrEP delivery in Kenyan family planning clinics, with participant enrollment from June 18, 2021, to May 18, 2023, and follow-up through February 02, 2024 (ClinicalTrials.gov: NCT04666792). The study population included sexually active HIV–negative women aged ≥15 years at elevated HIV risk per Kenyan PrEP guidelines. Participants were offered standard-of-care oral PrEP with the option to decline and followed quarterly for 12 months with assessments of HIV status, sexual behavior, and PrEP use. Urine samples were batch tested for *Neisseria gonorrhoeae* and *Chlamydia trachomatis* using the GeneXpert CT/NG real-time polymerase chain reaction nucleic acid amplification test assay. The primary exposure was self-reported PrEP initiation and PrEP use consistency through 6 months, categorized as never used PrEP, inconsistently on PrEP, or consistently on PrEP. Multivariable modified Poisson generalized estimating equation (GEE) models with robust standard errors were used to estimate associations between PrEP use and incident STI; clinic-level intracluster correlation coefficients were negligible. The secondary outcomes were incident HIV infection and sexual behaviors, which included condomless sex at last sex, sex with any new partners in the past 3 months, and multiple sex partners. HIV testing was performed at each scheduled visit, at enrollment, and 1, 3, 6, 9, and 12 months following the Kenya national HIV testing algorithm, using Determine HIV-1/2 and Fast Response test kits. All models for the primary outcome were adjusted for baseline covariates determined apriori as potential confounders, which included age, STI diagnosis at enrollment, any contraceptive use, number of sexual partners (categorized as any more than one sexual partner), education status, marital status, last partner HIV status, any transactional sex in 3 months pre-enrollment, and clinic site.

Among 650 women enrolled, 60.0% (389) initiated PrEP at baseline and 14.6% (38/261) initiated post-enrollment. Median age was 26 years (IQR 23–30), 40% (262/650) were aged ≤24 years, and 67% (436/648) did not know their primary partner’s HIV status. At baseline, 11% (74/650) had an STI, including 9.9% (23/232) of consistent PrEP users, 9.2% (13/141) of inconsistent users, and 14.0% (38/277) of women who declined PrEP. During follow-up, 19.1% (114/597) had at least one STI diagnosis, with similar risk among women who initiated PrEP at baseline compared with those who declined (19.2% [68/354] versus 18.9% [46/244]; aRR 1.11, 95% CI 0.76–1.61; *p* = 0.580). Compared with non-PrEP users (12.7% [25/195]), STI risk was 6.0% (12/200) among consistent PrEP users (aRR 0.56, 95% CI 0.27–1.19; *p* = 0.130) and 16.5% (17/103) among inconsistent users (aRR 1.43, 95% CI 0.73–2.77; *p* = 0.290). Chlamydia accounted for 87.7% (100/114) of STI diagnoses. STI risk was higher among women aged ≤24 years (aRR 1.47, 95% CI 1.04–2.07; *p* = 0.029) and those with a baseline STI (aRR 2.96, 95% CI 2.12–4.14; *p* < 0.001). Four HIV infections occurred over 594 person-years (incidence 0.67, 95% CI 0.18–1.72 per 100 person-years), including three among women who declined PrEP (incidence 1.25, 95% CI 0.26–3.66 per 100 person-years). The main study limitation was oral PrEP use was assessed based on client self-report and not objectively through drug levels testing.

**Conclusions:**

In this prospective cohort study among African women at elevated risk for HIV, 60% initiated PrEP at baseline and 14.6% (38/261) post-enrollment. PrEP use was not associated with increased risk for STI diagnosis through one year of follow-up. HIV incidence was low overall, consistent with expanded PrEP availability in similar populations.

## Author summary

**Why was this study done?:**

- Oral preexposure prophylaxis (PrEP) effectively reduces the risk of HIV infection when used with sufficient adherence but does not protect against other STIs.
- Several studies have documented high rates of bacterial STIs among individuals initiating and using PrEP.
- The role of PrEP in driving the increased risk for STI acquisition among individuals using PrEP has been a source of substantial scientific and public health debate.
- While bacterial STI incidence among men using PrEP is well documented, data on bacterial STI incidence among women using PrEP remain limited.

**What did the researchers do and find?:**

- We conducted an observational cohort study among 650 HIV–negative Kenyan women aged ≥15 years at substantial risk for HIV who were eligible for PrEP and enrolled between June 2021 and February 2024 at five public-sector family planning clinics.
- Participants were offered standard-of-care oral PrEP and followed quarterly for 12 months with HIV testing, batched urine-based testing for *Chlamydia trachomatis* (*C. trachomatis*) and *Neisseria gonorrhoeae* (*N. gonorrhoeae*) using NAAT (GeneXpert CT/NG), and assessments of sexual behavior, PrEP use, and HIV risk according to Kenya national PrEP guidelines.
- Approximately 20% of women were diagnosed with *C. trachomatis* or *N. gonorrhoeae* during follow-up, with chlamydia accounting for >87% of infections; rates of bacterial STI diagnosis for these two common curable STIs did not meaningfully differ by PrEP use status.
- About 60% of women offered PrEP initiated use; no HIV infections occurred among women reporting consistent PrEP use, despite high levels of condomless sex and >78% of participants not knowing their primary partner’s HIV status. Seventy-five percent of all HIV infections (3/4) occurred among women who declined PrEP through 12 months of follow-up

**What do these findings mean?:**

- Women who used PrEP did not have a higher risk of getting an STI over the year compared with women who did not use PrEP.
- The frequency of curable STIs among African women eligible for PrEP was high through one year of follow-up and was similar for different categories of PrEP use.
- No HIV infection occurred in women reporting consistent PrEP use, but 75% of all new HIV infections occurred in women who declined to initiate PrEP, representing a missed opportunity for HIV prevention.
- The major study limitations are oral PrEP use was assessed based on client self-report and not objectively through drug levels testing, and data are from family planning clinics which may not be generalizable to women accessing other health service delivery settings.

## Abstract 3

In an observational cohort study of women at risk of acquiring HIV, David Mukasa and colleagues report that initiating HIV preexposure prophylaxis did not associate with an increased incidence of sexually transmitted infections.

## Introduction

The HIV epidemic continues to pose significant public health challenges, particularly in sub-Saharan Africa [1], where women bear a disproportionate burden of the disease [2]. At the same time, African women also face a disproportionate burden of sexually transmitted infections (STIs), including high rates of curable bacterial pathogens such as *Chlamydia trachomatis* and *Neisseria gonorrhoeae* [3]. Preexposure prophylaxis (PrEP) with daily oral emtricitabine-tenofovir disoproxil fumarate (FTC/TDF) is a highly effective and approved strategy to prevent HIV acquisition when taken with sufficient adherence [4–8]. New, proven longer-acting PrEP options that could help overcome individual-level challenges are also rapidly emerging [9,10]. Although the effectiveness of PrEP against HIV is well established [11–16], all current approved HIV PrEP options do not and were not expected to protect against bacterial STIs.

The burden of STIs is highest in low- and middle-income countries worldwide, and since the beginning of the HIV epidemic, the coexisting epidemics of bacterial STIs and HIV have been recognized in Africa [17–21]. Despite evidence that the increasing STI trends [22] predate the PrEP era [23], as the adoption of oral PrEP for HIV prevention has gradually increased, its role in driving the increased risk for STI infection among individuals using PrEP has become a source of substantial scientific and public health debate. Some studies have suggested that PrEP use may alter an individual’s perceived risk and sexual behavior, potentially increasing exposure to other STIs if condom usage declines among PrEP users—a phenomenon commonly referred to as risk compensation [24,25]. Conversely, individuals who accept PrEP may have a higher perceived risk of HIV and STIs and be more cautious in general, and the increased frequency of medical visits and counseling associated with PrEP could offset that greater overall risk of STIs. Understanding these dynamics is crucial for informing public health strategies and optimizing integrated strategies to prevent HIV and STI transmission.

We hypothesized that among African women at elevated risk for HIV, PrEP use would not be associated with the risk of incident diagnosis of *C. trachomatis* and/or *N. gonorrhoeae* during follow-up. The overall aim of this prospective cohort study was to examine the association between PrEP use and the risk of *C. trachomatis* and *N. gonorrhoeae* diagnosis among African women who were assessed to be at elevated risk for HIV based on the Kenya PrEP guidelines.

## Methods

### Study design

Between June 18, 2021, and February 02, 2024, we conducted a prospective observational cohort study nested within a large pragmatic oral PrEP implementation project (the FP Plus Project). Participants in the nested cohort were enrolled between June 18, 2021, and May 18, 2023, with follow-up continuing through February 02, 2024. The details of the parent study protocol and methods are published elsewhere [26]. Briefly, the FP Plus Project was a stepped-wedge cluster randomized trial to integrate oral PrEP delivery in 12 public health family planning clinics in Kenya (ClinicalTrials.gov: NCT04666792). The overall goal of the parent stepped-wedge project was to catalyze scale-up of integrated oral PrEP delivery within public FP clinics using existing infrastructure and staff. The program was built for all HIV–negative women 15–49 years accessing the participating clinics. The intervention for the stepped-wedge design included technical support to the clinics and training of FP clinic staff to build their capacity to deliver integrated PrEP services as part of routine services in FP clinics. Within the large Stepped-wedge programmatic project, we enrolled a nested observational cohort of women not living with HIV but assessed by the FP clinic staff to be at elevated risk for HIV and eligible for PrEP. The overall goal of the nested cohort was to measure detailed individual-level outcomes on HIV prevention behavior, and STI burden, incident HIV, and PrEP use patterns. Both women who initiated PrEP and those eligible for PrEP but who chose not to initiate PrEP were eligible to be enrolled and followed up for 12 months. This study adheres to the Strengthening the Reporting of Observational Studies in Epidemiology (STROBE) guideline (S1 Checklist).

### Study population and setting

We enrolled 650 sexually active women who were not living with HIV and were accessing family planning services in the Kisumu area of Kenya, a region with an HIV prevalence of 28% among young women. Eligibility for the observational cohort was based on being ≥15 years old, sexually active, able and willing to provide consent for follow-up in the cohort, not known to live with HIV, and having at least one behavior and epidemiological factor defined by the Kenya PrEP guidelines to indicate a substantial ongoing risk of acquiring HIV. These behavior and epidemiological factors are the same indicators for PrEP eligibility defined by the Kenya PrEP guidelines and include: a) inconsistent condom use; b) having a sex partner(s) who is high risk and whose HIV status is unknown; c) engaging in transactional sex; d) ongoing intimate partner violence and gender-based violence; e) recent bacterial STI; f) recurrent use of post-exposure prophylaxis; g) recurrent sex under the influence of alcohol/recreational drugs; and h) injection drug use with shared needles and/or syringes [27]. At recruitment, all women were offered oral PrEP for HIV prevention by the Ministry of Health (MOH) staff as part of the standard of care. Within the large PrEP program, women assessed to be eligible for PrEP by the MOH staff were offered same-day PrEP initiation as part of the standard of care. Nearly 96% of all those who initiated PrEP started it on the first encounter they were deemed eligible for PrEP. Similarly, women eligible and interested in participating in the cohort were typically enrolled on the same day they were first assessed to be eligible for PrEP. PrEP medications and HIV testing kits were provided by the Kenya national stock of antiretrovirals from the Kenya Medical Supply Authority (KEMSA) as part of the national PrEP scale-up program. PrEP has regulatory and normative guidance sanctions in Kenya and is defined as part of standard care [28–37]. PrEP was provided at initiation, at one month, and quarterly, with the last refill provided 12 months after initiation.

### Facility selection

The nested cohort study was conducted at five of the 12 family planning clinics participating in a large stepped-wedge programmatic project in Kisumu County, Kenya. Clinic selection for the large Stepped-wedge PrEP program was based on high client volume, readiness to participate, catchment area to capture the diversity that represents the geographic, economic, and demographic characteristics of all women accessing family planning services, and breadth among the clinic staff. For the nested cohort, the five clinics were all high-volume clinics selected from the 12 clinics participating in the stepped-wedge program, based on feasibility to efficiently enroll and space availability to conduct research procedures.

### Follow-up and data collection

Enrolled women had research visits at enrollment and 1, 3, 6, 9, and 12 months, with HIV testing at each visit. HIV testing was performed in line with the Kenya national HIV testing algorithms, using Determine HIV-1/2 and Fast Response test kits. At each scheduled visit, structured questionnaires were administered to obtain demographics, PrEP use status, HIV risk, contraception method, and HIV prevention methods use. Urine samples were tested for *N. gonorrhoeae* and *C. trachomatis* using the GeneXpert CT/NG real-time PCR assay (Cepheid, CA, USA) for all participants at baseline and for a randomly selected subset at 3, 6, and 12 months due to logistical constraints. With additional funding, 83%–87% of archived urine samples from randomly selected participants were tested, and 91.8% of women had at least one or more follow-up STI test results. This ensured that sample sizes remained close to the calculated minimum and minimized anypotential impact on the analyses. In addition, the sample included all participants aged ≥15 and all who had a baseline infection. At each visit participants received comprehensive counseling on HIV and STI risk reduction, including provision of condoms and testing for HIV in accordance with national guidelines. Within the large PrEP program, PrEP delivery was done by MOH clinic staff, and PrEP was provided to clients including cohort participants for free as part of standard of care services. At the end of the study, cohort participants using PrEP continued to receive PrEP services at the clinics as part of standard of care services. All research procedures for the cohort activities were conducted by study-dedicated staff.

### Measurement of exposure of interest and outcome

**Fig 1:**
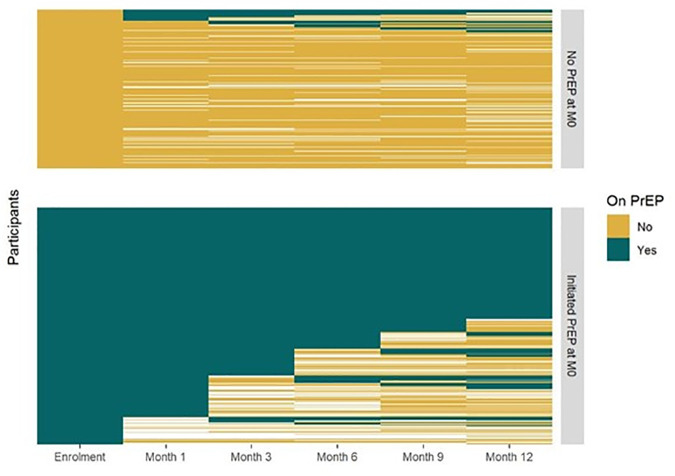
Preexposure prophylaxis use categorization (PrEP initiation at enrollment). Figure depicts PrEP usage patterns over 12 months, with assessments at each study visit: enrollment, month 1, and quarterly thereafter. Each horizontal line represents one participant, color-coded by PrEP use status (green = yes, gold = no, white = missing data). Figure categorizes participants by PrEP initiation at enrollment: 389 (60%) initiated. Each row represents an individual participant and each column a study visit month. The figure visualizes individual repeated measures; due to the large participant-by-visit structure, these underlying values are not interpretable in table form. The heat map displays temporal patterns in PrEP initiation, continuation, and switching patterns.

**Fig 2:**
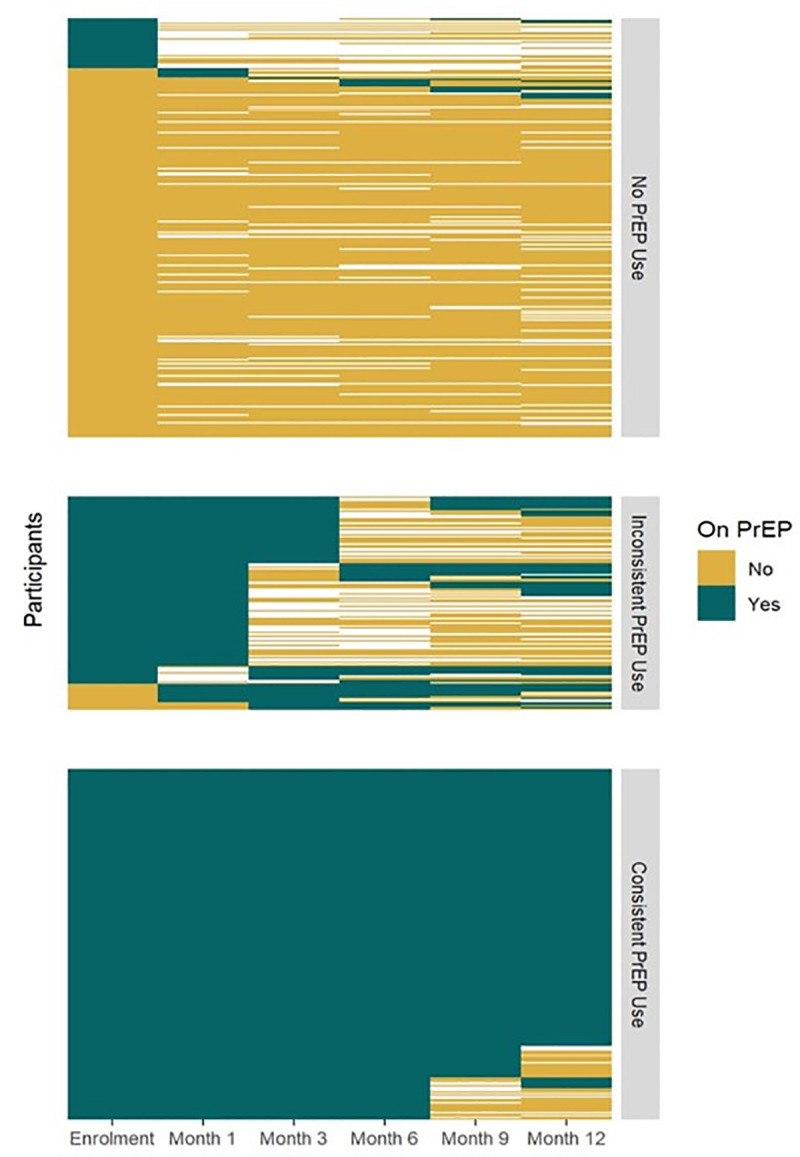
Preexposure prophylaxis use categorization (PrEP use through M6 (3 categories)). Figure depicts PrEP usage patterns over 12 months, with assessments at each study visit: enrollment, month 1, and quarterly thereafter. Each horizontal line represents one participant, color-coded by PrEP use status (green = yes, gold = no, white = missing data). Figure categorizes participants by PrEP use through M6: non-users (if initiated, did not refill; 277 [43%]), inconsistent users (≥1 refill, with gaps; 141 [22%]), or as consistent users (*n* = 232 [36%]). Each row represents an individual participant, and each column a study visit month. The figure visualizes individual repeated measures; due to the large participant-by-visit structure, these underlying values are not interpretable in table form. The heat map displays temporal patterns in PrEP initiation, continuation, and switching patterns.

Study participation required individuals to be eligible for oral PrEP based on the Ministry of Health PrEP guidelines, and both women who accepted and those who declined PrEP were eligible to be enrolled. The primary PrEP exposure was ascertained through both self-reported quantitative questionnaires on PrEP use and validated with clinic visit attendance and PrEP pharmacy refill records. PrEP exposure status was categorized in two ways to better triangulate PrEP exposure classification throughout the 12 months period (Figs 1 and 2). First, the primary exposure of interest was categorized as a binary variable based on PrEP initiation status ascertained at enrollment (i.e., initiated or not). Second, given that PrEP use is dynamic, and users may cycle on and off PrEP, and those who initiate may go off PrEP just after initiation, conditioned on baseline PrEP initiation status, we also classified PrEP use exposure based on consistency of PrEP use during the first 6 months since enrollment with three categories: never used PrEP at all, inconsistently on PrEP, and consistently on PrEP. In the primary analysis with the binary PrEP use exposure, the outcome was the number of etiological STI diagnoses of *C. trachomatis* or *N. gonorrhoeae* during the 12-month follow-up. For the second PrEP exposure variable based on the consistency of PrEP use through 6 months, the outcome was based on the number of etiological STI diagnoses of *C. trachomatis* or *N. gonorrhoeae* ascertained at months 6 and 12. For logistical reasons, real-time etiological testing for chlamydia and gonorrhea was conducted only at baseline. Participants diagnosed with chlamydia and gonorrhea at baseline were given their results and received treatment per the Ministry of Health standard of care and were also provided with expedited partner STI testing and therapy services. During follow-up period, only standard-of-care syndromic diagnosis and treatment were done, but at each visit, urine was collected and archived. Thus, testing for urine samples collected at months 3, 6, and 12 was conducted after the study ended and those results were not returned to study participants. Tenofovir diphosphate levels in dried blood spots among a subset of clients on oral PrEP were not available at time of analysis of the cohort and are not included in the current study. The secondary outcomes were incident HIV infection and follow-up sexual behaviors, which included condomless sex at last sex, sex with any new partners in the past 3 months, and multiple sex partners. HIV testing was performed at each scheduled visit, at enrollment, and 1, 3, 6, 9, and 12 months following the Kenya national HIV testing algorithm, using Determine HIV-1/2 and Fast Response test kits. Sexual behavior outcomes were ascertained through questionnaires administered at enrollment and 3, 6, 9, and 12 months.

### Statistical analysis

The sample size was calculated for the cohort study to investigate the association between PrEP use and the risk of sexually transmitted bacterial infections. We aimed to detect a relative risk (RR) of 1.5 when the probability of STI infection among women who declined PrEP use is 0.2 and with *α* = 0.05 and *β* = 0.20. The estimated sample size per group was 294 (a minimum total sample size of 588). 650 were enrolled to account for potential 10% attrition. The sample size was calculated using SAS version 9.4 (SAS Institute, Cary, NC). We used stratified sampling based on the primary exposure status to ensure an equal sample size in both the exposed and unexposed groups, though the exposed group in the study was slightly larger than the unexposed group. The primary hypothesis was that PrEP use is associated with the risk of incident *C. trachomatis* and/or *N. gonorrhoeae* diagnosis. A prospective statistical analysis plan was finalized prior to data analysis (Statistical Analysis Plan in S2 File). Analytic methods were guided by the study objectives, existing literature, and established epidemiologic principles. Analyses performed, including adjustments and sensitivity analyses, are explicitly described. Analytic refinements made during manuscript revision in response to peer review and the rationale included refining covariate adjustment to strengthen epidemiologic validity, assess robustness through sensitivity analyses, and improve transparency regarding missing data, clustering, and analytic assumptions.

Descriptive statistics were used to summarize sociodemographic, PrEP use status, and sexual behaviors. Categorical variables were summarized as frequencies and percentages, and non-normal continuous variables as medians and interquartile ranges. We used modified Poisson generalized estimating equation (GEE) models with robust standard errors with a working independence correlation matrix to estimate the RR and 95% confidence intervals (95% CIs) of infection with chlamydia or gonorrhoeae during follow-up by self-reported PrEP status [38]. GEE accounted for repeated observations per woman and clustering by clinic. Notably, clinic-level intracluster correlation coefficients were minimal to undetectable (0–0.0015), indicating negligible within-clinic correlation.

Pre-planned subgroup analyses were conducted within age categories and among those with STI diagnosis or treatment in 6 months pre-enrollment, STI at enrollment, and any contraceptive use at enrollment. As appropriate all models were adjusted for baseline covariates determined apriori potential confounders based on Directed Acyclic Graphs principles, which included age ≤24, STI diagnosis at enrollment, any contraceptive use, more than one sexual partner, education status, marital status, last partner HIV status, any transactional sex in 3 months pre-enrollment, and clinic site, all selected a priori. The adjusted models for the association between the primary exposure and secondary sexual behavioral outcomes included age, marital status, education status, and clinic site. Age was dichotomized at 24 years to distinguish young adults (15–24 years), a population that bears the greatest burden of HIV and STI in sub-Saharan Africa and is consistently identified in the literature as having lower PrEP awareness, uptake, and adherence and higher HIV incidence compared with older adults [2,39–41]. This cut-point aligns with definitions used by UNAIDS and CDC to characterize adolescents and young adults in HIV prevention research. All participants, including those with a baseline STI diagnosis, were included in this model, but only subsequent STI diagnoses were considered as events in the analysis. Three STI outcome measurement time points (i.e., 3, 6, and 12 months) were included in the primary analysis models based on the binary baseline PrEP initiation exposure.

For models using PrEP use exposure defined by PrEP consistency through 6 months (multi-category exposure); only STI outcome measurements ascertained at 6 and 12 months were included in the analysis. Behavioral outcomes were likewise evaluated at 3-, 6-, and 12-month visits to align with the 3-month recall period for these measures. We used a complete case analysis approach since the proportion of missing data was low (<1% for most covariates). Levels of missingness did not differ meaningfully by PrEP initiation status or by STI outcome categories. Variables with unreliable reporting (e.g., income) were excluded from adjusted analyses, and indeterminate STI test results were excluded from the analysis. Secondary outcomes included pathogen-specific STI diagnoses (*C. trachomatis* and *N. gonorrhoeae*), longitudinally assessed sexual behavior measures (condomless sex at last sex, new sexual partners in the past 3 months, and transactional sex), and HIV incidence. These analyses were conducted to provide contextual insight and are interpreted as secondary and exploratory hypothesis-generating. HIV incidence was estimated by dividing the number of new HIV infections by the accrued person-time of follow-up, overall and by PrEP status, with 95% CIs calculated using the Poisson exact method. Statistical analysis was performed using R version 4.4.1 and SAS ver. 9.4 (SAS Institute, Cary, NC).

### Ethics approval

The protocol, implementation plan, informed consent forms, data collection tools, and patient education materials were reviewed and approved by the University of Washington Human Subjects Review Committee and the Kenyatta National Hospital-University of Nairobi Ethical Review Committee (ClinicalTrials.gov: NCT04666792). All participants provided written informed consent for all research procedures not related to routine care. Participants received $5 as compensation reimbursement for their time and transportation expenses to participate in the study procedures for study visits.

## Results

### Participant general characteristics

**Fig 3:**
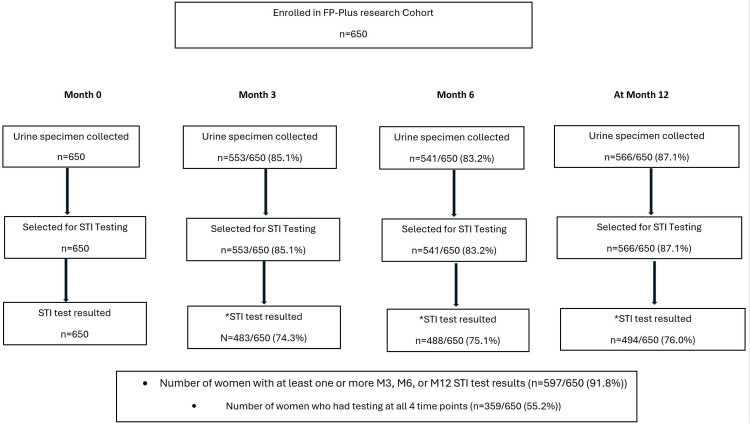
Flow diagram of study participants and sexually transmitted infections testing results received (Patient ID level). *Some results were indeterminate/inconclusive after testing. Numbers shown for participants with follow-up STI testing include only those with valid (non-indeterminate) results; indeterminate tests were excluded from outcome analyses.

A total of 650 participants were enrolled, and follow-up over the 12-month period was variable (Fig 3).

The median age (IQR) was 26 (23–30) years, and 40% (262/650) were ≤24 years, 81% (526/649) were married, 46% (296/650) had a primary or lower education level, 50% (282/568) had no personal income, and 85% (553/649) reported using at least one contraception method (Table 1). The most frequently used contraception methods at baseline were injectables at 31% (204/649), implants at 22% (143/649), and oral pills at 19% (123/649), as self-reported by the women. Overall, at enrollment, 67% (436/648) of the study participants did not know the HIV status of their primary sexual partner, and the HIV status of the person they last had sex with was unknown in 78% (504/645) (Table 1).

**Table 1:** Baseline characteristics stratified by baseline oral preexposure prophylaxis (PrEP) initiation status.

Covariate	Total (*n* = 650)		No PrEP at baseline (*n* = 261)		Initiated PrEP at baseline (*n* = 389)	
	*n*/*N* or Median	% or Q1–Q3	*n*/*N* or Median	%	*n*/*N* or Median	%
**Clinic site**						
A	197/650	(30.3%)	70/261	(26.8%)	127/389	(32.7%)
B	101/650	(15.5%)	51/261	(19.5%)	50/389	(12.9%)
C	129/650	(19.9%)	80/261	(30.7%)	49/389	(12.6%)
D	188/650	(28.9%)	48/261	(18.4%)	140/389	(35.9%)
E	35/650	(5.4%)	12/261	(4.6%)	23/389	(5.9%)
**Age (years)**	26	(23-30)	25	(22-29)	26	(23–31)
**Age (categories)**						
15–24	262/650	(40.3%)	122/261	(46.7%)	140/389	(36.0%)
25–35	337/650	(51.8%)	128/261	(49.1%)	209/389	(53.7%)
>35	51/650	(7.9%)	11/261	(4.2%)	40/389	(10.3%)
**Education level**						
Primary and below	296/650	(45.5%)	102/261	(39.0%)	194/389	(49.9%)
Completed secondary	179/650	(27.5%)	74/261	(28.4%)	105/389	(27.0%)
Attended post-secondary	175/650	(27.0%)	85/261	(32.6%)	90/389	(23.1%)
**Marital status**						
Not married	123/649	(19%)	42/260	(16%)	81/389	(21%)
Married	526/649	(81%)	218/260	(84%)	308/389	(79%)
**Personal income**						
No income	282/568	(49.6%)	124/242	(51.3%)	158/326	(48.5%)
1–5,000 Ksh	112/568	(19.7%)	46/242	(19.0%)	66/326	(20.2%)
5,001–10,000 Ksh	93/568	(16.4%)	36/242	(14.9%)	57/326	(17.5%)
>10,000 Ksh	38/568	(6.7%)	18/242	(7.4%)	20/326	(6.1%)
Declines to answer	43/568	(7.6%)	18/242	(7.4%)	25/326	(7.7%)
**Family planning (FP) method**						
Any FP method	553/649	(85%)	211/260	(81%)	342/389	(88%)
Injectable	204/649	(31%)	69/260	(27%)	135/389	(35%)
IUCD	20/649	(3.1%)	8/260	(3.1%)	12/389	(3.1%)
Implant	143/649	(22%)	44/260	(17%)	99/389	(25%)
Oral Contraceptive Pills	123/649	(19%)	48/260	(18%)	75/389	(19%)
Condoms	65/649	(10%)	42/260	(16%)	23/389	(5.9%)
**Any STI at baseline**	74/650	(11%)	33/261	(13%)	41/389	(11%)
**CT+ at baseline**	64/650	(9.8%)	31/261	(12%)	33/389	(8.5%)
**NG+ at baseline**	25/650	(3.8%)	11/261	(4.2%)	14/389	(3.6)%
**Primary partner HIV status**						
Negative	97/648	(15%)	48/260	(18%)	49/388	(13%)
Positive	14/648	(2.2%)	1/260	(0.4%)	13/388	(3.4%)
Unknown	436/648	(67%)	167/260	(64%)	269/388	(69%)
No primary partner	101/648	(16%)	44/260	(17%)	57/388	(15%)
**Last partner HIV status**						
Negative	120/645	(19%)	51/259	(20%)	69/386	(18%)
Positive	21/645	(3.3%)	1/259	(0.4%)	20/386	(5.2%)
Unknown	504/645	(78%)	207/259	(80%)	297/386	(77%)
**Multiple sexual partners (>1)**	81/649	(12.5%)	22/260	(8.5)	59/389	(15.2%)
**Any new partners (within the past 3 months)**	62/645	(9.6%)	17/259	(6.6%)	45/386	(12%)
**Any transactional sex (within the past 3 months)**	109/645	(16.9%)	46/259	(17.8%)	63/386	(16.3%)
**Condomless sex at last sex**	609/649	(93.8%)	245/260	(94.2%)	364/389	(93.6%)

*Statistics in Table 1 are *n*/*N* and (%) or median and (interquartile range); PrEP, preexposure prophylaxis; CT, *Chlamydia trachomatis*; NG, *Neisseria gonorrhoeae*; FP, family planning; IUCD, intrauterine contraceptive device.

### Oral PrEP uptake and use patterns

The heat map displaying the classification and patterns of reported PrEP use is presented in Fig 1 and 2. Overall, of 650 women, 60% (389/650) accepted PrEP while 261 declined PrEP at baseline. During follow- up, 14.6% (38/261) of women who initially declined subsequently started PrEP post-enrollment, translating into a total of 427 of 650 (65.7%) who ever initiated PrEP during the study period. Considering PrEP use patterns in the first 6 months, 42.6% (277/650) of women never started PrEP at all, 21.7% (141/650) inconsistently used PrEP while 35.7% (232/650) reported consistent PrEP use. Among women who initiated PrEP at baseline, 14.7% (57/389) discontinued PrEP but later restarted. Among those who reported inconsistent PrEP use, 36.2% (51/141) had only one refill visit after starting PrEP.

### Oral PrEP initiation, PrEP use consistency, and STI diagnosis through 12 months

During the 12-month follow-up, 608 women had urine collected for STI testing on at least one follow-up visit (i.e., 3, 6, or 12), of whom 597 had at least one NAAT test result (Fig 3). Among women with at least one NAAT result (*n* = 597), 19.1% (114) had at least one STI diagnosis: 19.2% (68/354) among women who initiated PrEP at baseline compared to 18.9% (46/244) among women who declined PrEP (adjusted relative aRR:1.11, 95%CI: 0.76–1.61; *p* = 0.580 (Tables 2 and S3). Overall, 11% (74/650) had an STI diagnosis at baseline: 9.9% (23/232) of those who consistently used PrEP, 9.2% (13/141) of those who inconsistently used PrEP, and 14% (38/277) of those who declined PrEP (S1 Table). Similarly, the frequency of any STI diagnosis was not different based on the reported consistency of PrEP use through 6 months: any STI was diagnosed in 6.0% (12/200) of women who were consistently on PrEP (aRR: 0.56, 95%CI: 0.27–1.19; *p* = 0.130), 16.5% (17/103) of women who were inconsistently on PrEP (aRR: 1.43, 95%CI: 0.73–2.77; *p* = 0.290) compared to 12.7% (25/197) of women who never used PrEP at all during follow-up (S2 Table). Chlamydia accounted for 87.7% (100/114) of the women who received at least one STI diagnosis (Table 2). The overall effect was consistent with etiology-specific rates for chlamydia and gonorrhea diagnoses (S2 Table).

**Table 2:** STI diagnosis through 12 months by oral PrEP initiation status at enrollment.

		Rate of STI		Rate ratio of STI			
Outcome	Level	No PrEP at enrollment	Initiated PrEP at enrollment	Initiated PrEP relative to did not			
		*n*/*N* (%)	*n*/*N* (%)	RR	*p*-value	aRR^1^	*p*-value
**Overall**							
Any STI		46/244 (18.9%)	68/354 (19.2%)	1.02 (0.73–1.43)	0.910	1.11 (0.76–1.61)	0.580
Chlamydia		42/244 (17.2%)	58/354 (16.4%)	0.95 (0.66–1.37)	0.790	1.05 (0.70–1.58)	0.800
Gonorrhea		8/244 (3.3%)	14/351 (4.0%)	1.22 (0.52–2.86)	0.650	1.27 (0.48–3.32)	0.630
**Subgroups: Age**							
Any STI	≤24	29/112 (25.9%)	29/118 (24.6%)	0.95 (0.61–1.49)	0.820	0.98 (0.57–1.71)	0.960
	>24	17/132 (12.9%)	39/236 (16.5%)	1.28 (0.75–2.18)	0.360	1.27 (0.71–2.30)	0.420
Chlamydia	≤24	27/112 (24.1%)	27/118 (22.9%)	0.95 (0.59–1.52)	0.830	0.97 (0.54–1.75)	0.910
	>24	15/132 (11.4%)	31/236 (13.1%)	1.16 (0.65–2.07)	0.630	1.07 (0.57–2.04)	0.830
**Subgroups: STI Dx or Tx in 6 months pre-enrollment**							
Any STI	No	39/210 (18.6%)	56/318 (17.6%)	0.95 (0.65–1.37)	0.780	0.99 (0.66–1.50)	0.960
	Yes	7/34 (20.6%)	12/36 (33.3%)	1.62 (0.71–3.71)	0.260	1.82 (0.42–7.84)	0.420
Chlamydia	No	35/210 (16.7%)	47/318 (14.8%)	0.89 (0.59–1.33)	0.560	0.92 (0.59–1.45)	0.730
	Yes	7/34 (20.6%)	11/36 (30.6%)	1.48 (0.64–3.47)	0.360	1.67 (0.35–7.98)	0.520
**Subgroups: STI at enrollment**							
Any STI	No	29/213 (13.6%)	50/316 (15.8%)	1.16 (0.76–1.78)	0.490	1.22 (0.75–1.99)	0.420
	Yes	17/31 (54.8%)	18/38 (47.4%)	0.86 (0.54–1.39)	0.550	—	—
Chlamydia	No	26/213 (12.2%)	40/316 (12.7%)	1.04 (0.65–1.65)	0.880	1.15 (0.68–1.95)	0.610
	Yes	16/31 (51.6%)	18/38 (47.4%)	0.92 (0.56–1.50)	0.730	—	—
**Subgroups: Any contraceptive use at enrollment**							
Any STI	No	4/48 (8.3%)	14/44 (31.8%)	3.82 (1.33–10.97)	0.0130	5.41 (1.55–18.93)	0.0082
	Yes	42/196 (21.4%)	54/310 (17.4%)	0.81 (0.57–1.17)	0.260	0.88 (0.59–1.32)	0.530
Chlamydia	No	3/48 (6.2%)	11/44 (25.0%)	4.00 (1.16–13.76)	0.028	4.97 (0.92–26.69)	0.062
	Yes	39/196 (19.9%)	47/310 (15.2%)	0.76 (0.52–1.12)	0.170	0.89 (0.57–1.38)	0.590

Output is suppressed for models which have very small numbers or otherwise do not converge.

^1^Covariates for the adjusted models in this table include: age less than 25, NG or CT positive at enrollment, any contraceptive use at enrollment, more than one sexual partner at enrollment, education status, marital status at enrollment, last partner HIV status, and any transactional sex in 3 months pre-enrollment, and clinic site; PrEP, preexposure prophylaxis; CT, *Chlamydia trachomatis*; NG, *Neisseria gonorrhoeae*; STI, sexually transmitted infections; Dx, diagnosis; Tx, treatment; M3, month 3; M6, month 6; M12, month 12.

### Sexual behaviors and association with oral PrEP use

**Fig 4:**
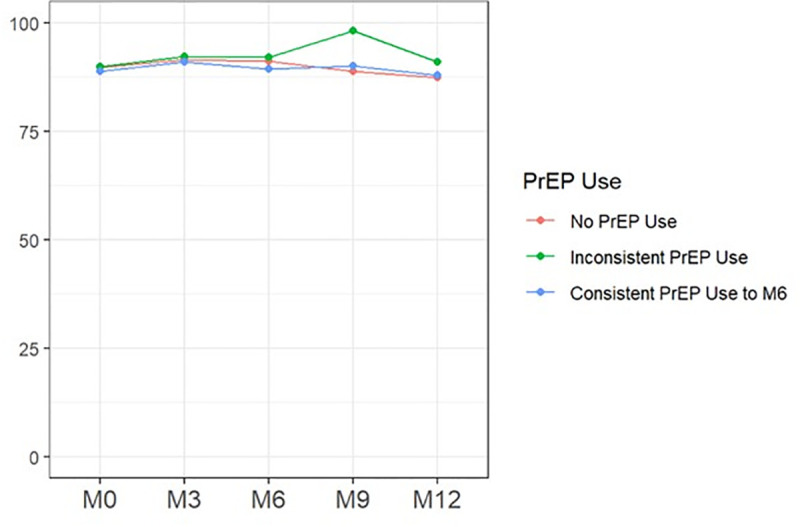
Percent of participants reporting condomless sex at last sex. The percentage of participants reporting condomless sex at last sexual encounter was assessed at baseline (M0) and at months 3(M3), 6 (M6), 9 (M9), and 12 (M12). Estimates are stratified by PrEP use classification: no PrEP use, inconsistent PrEP use, and consistent PrEP use through month 6. Values represent the proportion of respondents within each group reporting condomless sex at each time point.

Fig 4 shows patterns in the proportion of women reporting condomless sex at the last sex encounter by PrEP use status.

Overall, the frequency of condomless sex was high in the study population starting at baseline and remained high during the 12-month follow-up. Specifically, the proportion of women reporting condomless sex at the last sex encounter at baseline, 6, and 12 months was 89% (576/644), 90% (491/543), and 88% (489/554), respectively, which was not different between women who initiated versus those who declined PrEP at baseline (aRR: 0.99, 95% CI 0.73–1.35; *p* = 0.958). In contrast, sex with any new partners in the past 3 months (aRR: 1.54, 95% CI 1.01–2.37; *p* = 0.047) and multiple sex partners (aRR: 1.05, 95% CI 1.01–1.09; *p* = 0.0128) were reported more frequently among women who initiated compared to those who declined PrEP.

Similarly, during the 12 months of follow-up, there was no association between frequency of condomless sex at the last sex and PrEP use consistency through 6 months. Compared to women who never used PrEP at all during follow-up, the RR estimates were (aRR: 1.02, 95%CI: 0.73–1.43; *p* = 0.9138) for women consistently on PrEP and (aRR: 0.77, 95%CI: 0.53–1.12; *p* = 0.1688) for women inconsistently on PrEP. On the other hand, PrEP use through 6 months was associated with any new sex partners within the past 3 months: (aRR: 1.55, 95%CI: 0.99–2.43; *p* = 0.0538) for women consistently on PrEP and (aRR: 1.67, 95%CI: 1.01–2.74; *p* = 0.0447) for women inconsistently on PrEP. The association was also observed between PrEP use through 6 months and multiple sex partners: (aRR: 1.05, 95% CI: 1.01–1.10; *p* = 0.0157) for women consistently on PrEP and (aRR: 1.07, 95% CI: 1.019–1.12; *p* = 0.0056) for women inconsistently on PrEP compared to women who never used PrEP. Baseline associations between covariates and STI infection were examined to contextualize adjusted models but are presented in S3 Table to maintain focus on the primary exposure of interest (PrEP initiation).

### HIV incidence

Overall, a total of 594 person-years of follow-up were accrued with a median follow-up of 12.2 months (IQR: 10.8–12.9). Four women tested HIV–positive during the period, translating to an overall HIV incidence of 0.67 (95% CI: 0.18–1.72) cases per 100 person-years. The HIV incidence was 0.00 (95%CI: 0.00–1.58) cases per 100 person-years among women consistently on PrEP, 0.83 (95% CI: 0.02–4.61) cases per 100 person-years among women inconsistently on PrEP, and 1.25 (95% CI: 0.26–3.66) cases per 100 person-years for women never on PrEP during the first year of follow-up.

## Discussion

In this prospective cohort study among African women at substantial risk of HIV acquisition accessing family planning clinics, more than two-thirds used PrEP for HIV prevention at some point during the study period. STI diagnoses were highly concentrated among young women ≤24 years and those who had any STI at baseline, with 87% of the diagnoses due to chlamydia infection. Although STI diagnoses were high, the proportion of participants who had any STI diagnosis was relatively lower compared to what was reported in a randomized controlled trial on doxycycline postexposure prophylaxis (PEP) for cisgender women in the same setting [42]. The differences may be attributed to differences in the study designs, outcome definition, and inclusion criteria. The latter was an intervention study with more restrictive inclusion criteria for participants among women 18–30 years of age who were receiving PrEP against HIV and included Treponema pallidum in the outcome definition [42]. Overall, the one-year HIV incidence in the study population was 0.67 per 100 person-years, and no HIV acquisition occurred in women who reported consistently high adherence to PrEP.

Over the past two decades, the burden of bacterial STIs has increased substantially, coinciding with population-level implementation of emerging HIV prevention options, including oral PrEP and treatment as prevention. A systematic review and meta-analysis of the global epidemiology of STIs among persons using PrEP found a pooled prevalence of 24% for chlamydia, gonorrhea, or early syphilis at PrEP initiation and a pooled incidence rate of 72.2 per 100 person-years during the first three months of PrEP use [43]. In another study conducted among men who have sex with men, the proportion of participants diagnosed with any STI in the past 6 months was high among PrEP users compared to those who were not using PrEP (17.4% versus 8.4%) [44]. On the other hand, studies among female sex workers [45] and women who inject drugs [46] found no evidence and limited evidence for risk compensation, respectively. In sub-Saharan Africa, HIV transmission is predominantly through sexual exposure, and thus, it is not surprising to find a high STI burden in study populations with behavioral factors associated with an increased HIV risk. With the global rise in rates of STIs, the role of PrEP in driving changes in sexual behavior and increased risk for STI acquisition has been a source of substantial scientific and public health debate [47–49]. Some studies have suggested that PrEP use may alter an individual’s perceived risk and sexual behavior, potentially increasing exposure to other STIs if condom usage declines among PrEP users due to risk compensation [24,25]. On the contrary, PrEP users may be more cautious overall and consider themselves to be at a higher risk of HIV and STIs; the increased frequency of PrEP-related medical visits and counseling could offset this increased risk of STIs. Importantly, despite the high frequency of condomless sex and etiological diagnoses of infection with *C. trachomatis* or *N. gonorrhoeae* in this study population, we reassuringly found no evidence to suggest that PrEP use is associated with increased risk for STIs among African women at elevated risk for HIV accessing family planning clinics in Kenya.

Oral FTC/TDF PrEP is an effective, recommended, and impactful strategy for HIV prevention when taken with sufficient adherence. Even with self-reported adherence, a clear relationship was observed in this study between higher reported adherence and lower risk of HIV acquisition. In this study population, 78% of women did not know the HIV status of their sexual partners, and 75% of all new HIV acquisitions occurred in women who were offered but declined to initiate oral PrEP. The above results not only represent a missed opportunity but also highlight an urgent need to accelerate access to new PrEP choices for HIV prevention for people for whom daily oral PrEP is challenging or may not be the right prevention option for them. PrEP is an important prevention option for individuals at substantial risk for HIV, including sexual partners of people living with HIV who have not yet achieved full viral suppression. However, because oral PrEP and any currently approved PrEP option are not designed to prevent bacterial STIs, a potential increase in risk taking behavior among those using PrEP has the potential to increase exposure to STIs that can also offset benefits of PrEP. Thus, WHO has called for renewed efforts to provide integrated HIV and STI services [50].

This study included several secondary outcomes and exploratory analyses. These secondary findings should be interpreted cautiously and viewed as hypothesis-generating rather than definitive. Future studies with larger samples and confirmatory analytic designs are needed to validate these associations. There are several strengths and limitations to our study. The study’s strengths include a large prospective cohort of sexually active women, including those on PrEP as well as those at elevated risk of HIV but who declined oral PrEP, extensive etiological *N. gonorrhoeae* and *C. trachomatis* testing at multiple time points, and testing in real-world family planning clinic settings, which increases the representativeness of our study population. There are also several limitations to our study. First, this was an observational study, and groups were formed based on self-selection and self-reported oral PrEP use. Secondly, the urine GeneXpert CT/NG NAAT test is a practice testing strategy, particularly for large-scale STI screening programs, but it is not as sensitive for women as self-collected vaginal swab or provider-collected endocervical swabs. In addition, there was no testing for syphilis and Trichomonas vaginosis, and thus, there is a potential for underestimation of the overall STI prevalence. However, there is no impact on the primary question, as the non-testing syphilis or Trichomonas vaginosis applied uniformly across study exposure groups. Thirdly, the data is from family planning clinics, and this may reduce generalizability to other settings. Furthermore, women’s access to STI prevention advice in family planning clinic settings might have affected the outcome. Fourthly, PrEP use exposure was ascertained through a combination of visit attendance, PrEP prescription and dispensing data based on programmatic records extracted by the Ministry of Health staff, and client self-report PrEP use, which is prone to recall and social desirability bias. Fifthly, no objective measure of PrEP use was available, but the observation of a clear relationship between self-reported adherence and risk of HIV acquisition lends confidence to our findings. This was not an efficacy study, and it was not powered to test differences in HIV incidence between PrEP use categories. Recruitment into the nested study was not through random selection, and participants received IRB-approved compensation for their time, which could have introduced the potential for selection bias and reduced the generalizability of findings. Lastly, residual confounding is possible, particularly for covariates that were excluded from adjusted models due to concerns about measurement reliability. Despite these limitations, our large prospective data provides some of the most comprehensive empirical evidence on the association between PrEP use and the risk of bacterial STI infections.

In this large prospective cohort study among Kenyan women with provider-assessed elevated risk for HIV acquisition, 65.7% initiated PrEP. Despite high rates of condomless sex in this study population, we found no evidence to suggest that PrEP use is associated with increased risk for STIs among African women at elevated risk for HIV. No HIV acquisition occurred among women consistently on oral PrEP, but 75% of all new HIV acquisitions occurred in women who declined to initiate PrEP, representing a missed opportunity for HIV prevention.

## Supporting information

S1 Table Baseline characteristics stratified by preexposure prophylaxis (PrEP) initiation status. (DOCX)

S2 Table STI infection at 12 months by PrEP use consistency through month 6 since PrEP initiation (3 categories). (DOCX)

S3 Table Association between baseline covariates and STI diagnosis through 12 months. (DOCX)

S1 Checklist This checklist is licensed under the Creative Commons Attribution 4.0 International License (CC BY 4.0; https://creativecommons.org/licenses/by/4.0/). (DOC)

S1 File FP Plus Protocol_V3.0. PrEP-aring family planning clinics to streamline integration of HIV prevention services for young women in Kenya. The Family Planning plus HIV prevention (FP-Plus) Project.
(PDF)

S2 File FP Plus Statistical Analysis Plan_V1.0. (PDF)

S3 File S1 Supplementary materials. (DOCX)

## Abbreviations

FTC/TDF: emtricitabine-tenofovir disoproxil fumarate
GEE: generalized estimating equation
KEMSA: Kenya Medical Supply Authority
MOH: Ministry of Health
PEP: postexposure prophylaxis
PrEP: preexposure prophylaxis
RR: relative risk
STIs: sexually transmitted infections
STROBE: Strengthening the Reporting of Observational Studies in Epidemiology
