# Supplementary material for: Oral preexposure prophylaxis use and the risk of bacterial sexually transmitted infections and HIV among African women: A prospective observational cohort study
Source: PLoS Med. 2026 Mar 9;23(3):e1004962. doi: 10.1371/journal.pmed.1004962 (PMC13002101; doi:10.1371/journal.pmed.1004962)
Supplement: S1 Table — (DOCX) [file pmed.1004962.s001.docx]

**Supplementary Files**

**S1 Table: Baseline Characteristics Stratified by Preexposure Prophylaxis (PrEP) initiation status**

| **Covariate** | **Total (n=650)** | | **No PrEP Use**  **(n=277)** | | **Inconsistent PrEP Use**  **(n=141)** | | **Consistent PrEP Use**  **(n=232)** | |
| --- | --- | --- | --- | --- | --- | --- | --- | --- |
|  | **n/N or Median** | **% or Q1-Q3** | **n/N or Median** | **% or Q1-Q3** | **n/N or Median** | **% or Q1-Q3** |  | **% or Q1-Q3** |
| **Clinic Site** |  |  |  |  |  |  |  |  |
| A | 197/650 | (30.3%) | 76/277 | (27.4%) | 27/141 | (19.2%) | 94/232 | (40.5%) |
| B | 101/650 | (15.5%) | 51/277 | (18.4%) | 23/141 | (16.3%) | 27/232 | (11.6%) |
| C | 129/650 | (19.9%) | 78/277 | (28.2%) | 25/141 | (17.7%) | 26/232 | (11.2%) |
| D | 188/650 | (28.9%) | 63/277 | (22.7%) | 57/141 | (40.4%) | 68/232 | (29.3%) |
| E | 35/650 | (5.4%) | 9/277 | (3.3%) | 9/141 | (6.4%) | 17/232 | (7.4%) |
| **Age (years)** | 26 | (23-30) | 25 | (22-29) | 25 | (22-28) | 28 | (24-32) |
| **Age (categories)** |  |  |  |  |  |  |  |  |
| 15-24 | 262/650 | (40.3%) | 129/277 | (46.6%) | 64/141 | (45.4%) | 69/232 | (29.7%) |
| 25-35 | 337/650 | (51.8%) | 135/277 | (48.7%) | 70/141 | (49.7%) | 132/232 | (56.9%) |
| >35 | 51/650 | (7.9%) | 13/277 | (4.7%) | 7/141 | (4.9%) | 31/232 | (13.4%) |
| **Education level** |  |  |  |  |  |  |  |  |
| Primary and below | 296/650 | (46%) | 108/277 | (39%) | 62/141 | (44%) | 126/232 | (54%) |
| Completed secondary | 179/650 | (28%) | 80/277 | (29%) | 33/141 | (23%) | 66/232 | (28%) |
| Attended post-secondary | 175/650 | (27%) | 89/277 | (32%) | 46/141 | (33%) | 40/232 | (17%) |
| **Marital Status** |  |  |  |  |  |  |  |  |
| Not married | 123/649 | (19%) | 51/276 | (18%) | 33/141 | (23%) | 39/232 | (17%) |
| Married | 526/649 | (81%) | 225/276 | (82%) | 108/141 | (77%) | 193/232 | (83%) |
| **Personal income** |  |  |  |  |  |  |  |  |
| No income | 282/568 | (50%) | 132/257 | (51%) | 61/119 | (51%) | 89/192 | (46%) |
| 1 - 5,000 Ksh | 112/568 | (20%) | 45/257 | (18%) | 25/119 | (21%) | 42/192 | (22%) |
| 5,001 - 10,000 Ksh | 93/568 | (16%) | 41/257 | (16%) | 21/119 | (18%) | 31/192 | (16%) |
| > 10,000 Ksh | 38/568 | (6.7%) | 21/257 | (8.2%) | 5/119 | (4.2%) | 12/192 | (6.2%) |
| Declines to answer | 43/568 | (7.6%) | 18/257 | (7.0%) | 7/119 | (5.9%) | 18/192 | (9.4%) |
| **Family Planning (FP) Method** |  |  |  |  |  |  |  |  |
| Any FP method | 553/649 | (85%) | 225/276 | (82%) | 116/141 | (82%) | 212/232 | (91%) |
| Injectable | 204/649 | (31%) | 75/276 | (27%) | 49/141 | (35%) | 80/232 | (34%) |
| IUCD | 20/649 | (3.1%) | 8/276 | (2.9%) | 4/141 | (2.8%) | 8/232 | (3.4%) |
| Implant | 143/649 | (22%) | 52/276 | (19%) | 30/141 | (21%) | 61/232 | (26%) |
| Oral Contraceptive Pills | 123/649 | (19%) | 46/276 | (17%) | 25/141 | (18%) | 52/232 | (22%) |
| Condoms | 65/649 | (10%) | 44/276 | (16%) | 10/141 | (7.1%) | 11/232 | (4.7%) |
| **Any STI at baseline** | 74/650 | (11%) | 38/277 | (14%) | 13/141 | (9.2%) | 23/232 | (9.9%) |
| **CT+ at baseline** | 64/650 | (9.8%) | 34/277 | (12%) | 11/141 | (7.8%) | 19/232 | (8.2%) |
| **NG+ at baseline** | 25/650 | (3.8%) | 12/277 | (4.3%) | 5/141 | (3.5%) | 8/232 | (3.4%) |
| **Primary partner HIV status** |  |  |  |  |  |  |  |  |
| Negative | 97/648 | (15%) | 47/276 | (17%) | 22/141 | (16%) | 28/231 | (12%) |
| Positive | 14/648 | (2.2%) | 0/276 | (0%) | 4/141 | (2.8%) | 10/231 | (4.3%) |
| Unknown | 436/648 | (67%) | 189/276 | (68%) | 84/141 | (60%) | 163/231 | (71%) |
| No primary partner | 101/648 | (16%) | 40/276 | (14%) | 31/141 | (22%) | 30/231 | (13%) |
| **Last partner HIV status** |  |  |  |  |  |  |  |  |
| Negative | 120/645 | (19%) | 52/276 | (19%) | 32/139 | (23%) | 36/230 | (16%) |
| Positive | 21/645 | (3.3%) | 0/276 | (0%) | 6/139 | (4.3%) | 15/230 | (6.5%) |
| Unknown | 504/645 | (78%) | 224/276 | (81%) | 101/139 | (73%) | 179/230 | (78%) |
| **Multiple sexual partners (>1)** | 81/649 | (12.5) | 20/276 | (7.3) | 28/141 | (19.9) | 33/232 | (14.2) |
| **Any new partners (within the past 3 months)** | 62/645 | (9.6%) | 15/276 | (5.4%) | 20/139 | (14%) | 27/230 | (12%) |
| **Any transactional sex (within the past 3 months)** | 109/645 | (16.9) | 46/276 | (16.7) | 34/139 | (24.5) | 29/230 | (12.6) |
| **Condom used at last sex** | 68/644 | (11%) | 28/274 | (10%) | 14/139 | (10%) | 26/231 | (11%) |

*Statistics are n/N and (%) or median and (interquartile range); PrEP: Preexposure Prophylaxis; MO: Month Zero; CT: *Chlamydia trachomatis;* NG: Neisseria gonorrhoeae; FP: Family Planning; IUCD: Intrauterine Contraceptive Device.
