# Supplementary material for: Oral preexposure prophylaxis use and the risk of bacterial sexually transmitted infections and HIV among African women: A prospective observational cohort study
Source: PLoS Med. 2026 Mar 9;23(3):e1004962. doi: 10.1371/journal.pmed.1004962 (PMC13002101; doi:10.1371/journal.pmed.1004962)
Supplement: S2 Table — (DOCX) [file pmed.1004962.s002.docx]

**Supplementary Files**

**S2 Table: STI daignosis at 12 Months by PrEP use consistency through Month 6 since PrEP initiation (3 categories)**

| **Rate of STI** | | | | | **Rate ratio of STI** | | | | | | | |
| --- | --- | --- | --- | --- | --- | --- | --- | --- | --- | --- | --- | --- |
| **Outcome** | **Level** | **No PrEP Use** | **Inconsistent PrEP Use** | **Consistent PrEP Use** | **Inconsistent PrEP use**  **(relative to No PrEP use)** | | | | **Consistent PrEP use**  **(relative to No PrEP use)** | | | |
|  |  | **n/N (%)** | **n/N (%)** | **n/N (%)** | **RR** | **P-value** | **aRR^1^** | **P-value** | **RR** | **P-value** | **aRR^1^** | **P-value** |
| **Overall** | | | | | | | | | | | | |
| Any STI |  | 25/197 (12.7%) | 17/103 (16.5%) | 12/200 (6.0%) | 1.30 (0.73–2.31) | 0.370 | 1.43 (0.73–2.77) | 0.290 | 0.47 (0.24–0.92) | 0.027 | 0.56 (0.27–1.19) | 0.130 |
| Chlamydia |  | 21/197 (10.7%) | 16/103 (15.5%) | 9/200 (4.5%) | 1.46 (0.79–2.68) | 0.230 | 1.66 (0.82–3.39) | 0.160 | 0.42 (0.20–0.90) | 0.026 | 0.57 (0.24–1.34) | 0.190 |
| Gonorrhea |  | 4/197 (2.0%) | 1/103 (1.0%) | 4/200 (2.0%) | 0.48 (0.05–4.31) | 0.510 | 0.35 (0.03–3.61) | 0.380 | 0.99 (0.25–3.91) | 0.980 | 0.63 (0.11–3.61) | 0.600 |
| **Subgroups: Age** | | | | | | | | | | | | |
| Any STI | 15-24 | 14/85 (16.5%) | 10/44 (22.7%) | 3/57 (5.3%) | 1.38 (0.66–2.89) | 0.390 | 1.55 (0.66–3.66) | 0.320 | 0.32 (0.09–1.08) | 0.067 | 0.40 (0.10–1.53) | 0.180 |
|  | 25-49 | 11/112 (9.8%) | 7/59 (11.9%) | 9/143 (6.3%) | 1.21 (0.49–2.99) | 0.680 | 1.18 (0.37–3.82) | 0.780 | 0.64 (0.27–1.50) | 0.310 | 0.68 (0.25–1.86) | 0.460 |
| Chlamydia | 15-24 | 13/85 (15.3%) | 10/44 (22.7%) | 2/57 (3.5%) | 1.49 (0.70–3.16) | 0.300 | 1.67 (0.69–4.05) | 0.260 | 0.23 (0.05–1.00) | 0.050 | 0.29 (0.06–1.44) | 0.130 |
|  | 25-49 | 8/112 (7.1%) | 6/59 (10.2%) | 7/143 (4.9%) | 1.42 (0.51–3.97) | 0.500 | 1.48 (0.39–5.52) | 0.560 | 0.69 (0.25–1.85) | 0.460 | 0.83 (0.24–2.84) | 0.760 |
| **Subgroups: STI Dx or Tx in 6 months pre-enrollment** | | | | | | | | | | | | |
| Gonorrhea | No | 4/170 (2.4%) | 0/91 (0.0%) | 4/179 (2.2%) | — | — | — | — | 0.95 (0.24–3.77) | 0.940 | 0.70 (0.10–4.71) | 0.720 |
|  | Yes | 0/27 (0.0%) | 1/12 (8.3%) | 0/21 (0.0%) | — | — | — | — | 1.00 (0.75–1.33) | 1.000 | — | — |
| Chlamydia | No | 19/170 (11.2%) | 13/91 (14.3%) | 8/179 (4.5%) | 1.28 (0.66–2.48) | 0.470 | 1.45 (0.67–3.14) | 0.350 | 0.40 (0.18–0.89) | 0.025 | 0.57 (0.23–1.40) | 0.220 |
|  | Yes | 2/27 (7.4%) | 3/12 (25.0%) | 1/21 (4.8%) | 3.37 (0.59–19.41) | 0.170.20 | — | — | 0.64 (0.06–7.37) | 0.720 | — | — |
| **Subgroups: STI at enrollment** | | | | | | | | | | | | |
| Any STI | No | 18/174 (10.3%) | 14/92 (15.2%) | 12/181 (6.6%) | 1.47 (0.76–2.84) | 0.250 | 1.54 (0.73–3.24) | 0.250 | 0.64 (0.32–1.30) | 0.220 | 0.72 (0.32–1.58) | 0.410 |
|  | Yes | 7/23 (30.4%) | 3/11 (27.3%) | 0/19 (0.0%) | 0.90 (0.26–3.10) | 0.860 | — | — | — | — | — | — |
| Chlamydia | No | 14/174 (8.0%) | 13/92 (14.1%) | 9/181 (5.0%) | 1.76 (0.86–3.60) | 0.120 | 1.92 (0.86–4.30) | 0.110 | 0.62 (0.27–1.40) | 0.250 | 0.79 (0.31–1.96) | 0.600 |
|  | Yes | 7/23 (30.4%) | 3/11 (27.3%) | 0/19 (0.0%) | 0.90 (0.26–3.10) | 0.860 | — | — | — | — | — | — |
| **Subgroups: Any contraceptive use at enrollment** | | | | | | | | | | | | |
| Any STI | No | 2/38 (5.3%) | 6/19 (31.6%) | 2/17 (11.8%) | 6.00 (1.27–28.31) | 0.024 | — | — | 2.24 (0.32–15.83) | 0.420 | 9.15 (0.09–920.61) | 0.350 |
|  | Yes | 23/159 (14.5%) | 11/84 (13.1%) | 10/183 (5.5%) | 0.91 (0.46–1.78) | 0.770 | 0.91 (0.40–2.04) | 0.810 | 0.38 (0.18–0.77) | 0.0077 | 0.41 (0.18–0.90) | 0.027 |
| Chlamydia | No | 2/38 (5.3%) | 6/19 (31.6%) | 1/17 (5.9%) | 6.00 (1.27–28.31) | 0.024 | — | — | 1.12 (0.10–12.95) | 0.930 | — | — |
|  | Yes | 19/159 (11.9%) | 10/84 (11.9%) | 8/183 (4.4%) | 1.00 (0.48–2.06) | 0.990 | 1.06 (0.44–2.56) | 0.890 | 0.37 (0.16–0.82) | 0.014 | 0.44 (0.18–1.13) | 0.087 |
| *^Output is suppressed for models which have very small numbers or otherwise do not converge^*  *^1^* Covariates for the adjusted models in this table include: age less than 25, NG or CT positive at enrollment, any contraceptive use at enrollment, more than one sexual partner at enrollment, education status, marital status at enrollment, last partner HIV status, and any transactional sex in 3 months pre-enrollment, and clinic site | | | | | | | | | | | | |

For each outcome, the unadjusted and adjusted models used identical denominators, and no additional observations were excluded at the adjustment stage.
