# Supplementary material for: Oral preexposure prophylaxis use and the risk of bacterial sexually transmitted infections and HIV among African women: A prospective observational cohort study
Source: PLoS Med. 2026 Mar 9;23(3):e1004962. doi: 10.1371/journal.pmed.1004962 (PMC13002101; doi:10.1371/journal.pmed.1004962)
Supplement: S3 Table — (DOCX) [file pmed.1004962.s003.docx]

**S3 Table: Association between baseline covariates and STI diagnosis through 12 months**

| **Exposure** | **Level** | **n/N (%)** | **RR** | | | **p-value** | **aRR** | | | **p-value** | |
| --- | --- | --- | --- | --- | --- | --- | --- | --- | --- | --- | --- |
| **Outcome: Any STI** | |  | | |  |  |  | | | |  |
| Initiated PrEP at baseline | No | 46/244 (18.9%) | | | Reference |  | | Reference | | |  |
|  | Yes | 68/354 (19.2%) | | | 1.02 (0.73–1.43) | 0.910 | 1.11 (0.76–1.61) | | | | 0.580 |
| Age groups | ≤24 | 58/230 (25.2%) | | | 1.66 (1.19–2.30) | 0.0026 | 1.47 (1.04–2.07) | | | | 0.029 |
|  | >24 | 56/368 (15.2%) | | | Reference |  | | |  | |  |
| NG or CT positive^✝^ | No | 79/529 (14.9%) | | | Reference |  | Reference | | | |  |
|  | Yes | 35/69 (50.7%) | | | 3.40 (2.49–4.64) | <0.001 | 2.96 (2.12–4.14) | | | | <0.001 |
| Any contraceptive use^✝^ | No | 18/92 (19.6%) | | | Reference |  | Reference | | | |  |
|  | Yes | 96/506 (19.0%) | | | 0.97 (0.61–1.53) | 0.890 | 0.95 (0.59–1.53) | | | | 0.840 |
| Education Status | Primary and below | 45/276 (16.3%) | | | Reference |  | Reference | | | |  |
|  | Completed secondary | 36/159 (22.6%) | | | 1.39 (0.94–2.06) | 0.100 | 1.11 (0.75–1.65) | | | | 0.600 |
|  | Attended post-secondary | 33/163 (20.2%) | | | 1.24 (0.83–1.87) | 0.300 | 1.23 (0.80–1.88) | | | | 0.350 |
| More than 1 partner^✝^ | 0-1 | 97/526 (18.4%) | | Reference | |  | Reference | | |  | |
|  | >1 | 17/72 (23.6%) | | 1.28 (0.81–2.02) | | 0.290 | 1.05 (0.63–1.74) | | | 0.850 | |
| Marital status^✝^ | Not married | 25/103 (24.3%) | | Reference | |  | Reference | | |  | |
|  | Married | 89/495 (18.0%) | | 0.74 (0.50–1.10) | | 0.130 | 0.87 (0.57–1.35) | | | 0.540 | |
| Last partner HIV status | Negative | 17/113 (15.0%) | | Reference | |  | Reference | | |  | |
|  | Positive | 5/20 (25.0%) | | 1.66 (0.67–4.14) | | 0.280 | 2.14 (0.84–5.48) | | | 0.110 | |
|  | Unknown | 92/462 (19.9%) | | 1.32 (0.82–2.14) | | 0.250 | 1.30 (0.81–2.10) | | | 0.280 | |
| Any transactional sex^🞰^ | No | 91/495 (18.4%) | | Reference | |  | Reference | | |  | |
|  | Yes | 23/100 (23.0%) | | 1.25 (0.83–1.88) | | 0.280 | 1.06 (0.67–1.67) | | | 0.800 | |
| Clinic Site | A | 44/194 (22.7%) | | Reference | |  | Reference | | |  | |
|  | B | 22/98 (22.4%) | | 0.99 (0.63–1.56) | | 0.96 | 0.83 (0.50–1.40) | | | 0.490 | |
|  | C | 24/124 (19.4%) | | 0.85 (0.55–1.33) | | 0.49 | 0.77 (0.45–1.31) | | | 0.330 | |
|  | D | 22/149 (14.8%) | | 0.65 (0.41–1.04) | | 0.072 | 0.69 (0.42–1.12) | | | 0.130 | |
|  | E | 2/33 (6.1%) | | 0.27 (0.07–1.09) | | 0.067 | 0.29 (0.07–1.23) | | | 0.094 | |
| **Outcome: Chlamydia** | | | | | | | | | | | |
| Initiated PrEP at M0 | No | 42/244 (17.2%) | | Reference | |  | Reference | | |  | |
|  | Yes | 58/354 (16.4%) | | 0.95 (0.66–1.37) | | 0.790 | 1.05 (0.70–1.58) | | | 0.800 | |
| Age | 15-24 | 54/230 (23.5%) | | 1.88 (1.31–2.69) | | <0.001 | 1.65 (1.14–2.40) | | | 0.0086 | |
|  | 25-49 | 46/368 (12.5%) | | Reference | |  | Reference | | |  | |
| NG or CT positive^✝^ | No | 66/529 (12.5%) | | Reference | |  | Reference | | |  | |
|  | Yes | 34/69 (49.3%) | | 3.95 (2.83–5.50) | | <0.001 | 3.42 (2.40–4.88) | | | <0.001 | |
| Any contraceptive use^✝^ | No | 14/92 (15.2%) | | Reference | |  | Reference | | |  | |
|  | Yes | 86/506 (17.0%) | | 1.12 (0.66–1.89) | | 0.680 | 1.11 (0.63–1.94) | | | 0.720 | |
| Education Status | Primary and below | 38/276 (13.8%) | | Reference | |  | Reference | | |  | |
|  | Completed secondary | 30/159 (18.9%) | | 1.37 (0.88–2.13) | | 0.160 | 1.04 (0.67–1.61) | | | 0.850 | |
|  | Attended post-secondary | 32/163 (19.6%) | | 1.43 (0.93–2.19) | | 0.110 | 1.37 (0.87–2.17) | | | 0.180 | |
| More than 1 partner^✝^ | 0-1 | 85/526 (16.2%) | | Reference | |  | Reference | | |  | |
|  | >1 | 15/72 (20.8%) | | 1.29 (0.78–2.12) | | 0.320 | 1.06 (0.62–1.82) | | | 0.830 | |
| Marital status^✝^ | Not married | 21/103 (20.4%) | | Reference | |  | Reference | | |  | |
|  | Married | 79/495 (16.0%) | | 0.78 (0.51–1.21) | | 0.270 | 0.96 (0.60–1.53) | | | 0.860 | |
| Last partner HIV status | Negative | 17/113 (15.0%) | | Reference | |  | Reference | | |  | |
|  | Positive | 4/20 (20.0%) | | 1.33 (0.48–3.70) | | 0.590 | 1.81 (0.64–5.11) | | | 0.260 | |
|  | Unknown | 79/462 (17.1%) | | 1.14 (0.70–1.85) | | 0.610 | 1.16 (0.70–1.90) | | | 0.560 | |
| Any transactional sex^🞰^ | No | 79/495 (16.0%) | | Reference | |  | Reference | | |  | |
|  | Yes | 21/100 (21.0%) | | 1.32 (0.85–2.03) | | 0.220 | 1.05 (0.65–1.70) | | | 0.830 | |
| Clinic Site | A | 38/194 (19.6%) | | Reference | |  | Reference | | |  | |
|  | B | 21/98 (21.4%) | | 1.09 (0.68–1.77) | | 0.71 | 0.87 (0.50–1.52) | | | 0.630 | |
|  | C | 20/124 (16.1%) | | 0.82 (0.50–1.35) | | 0.44 | 0.73 (0.41–1.32) | | | 0.300 | |
|  | D | 19/149 (12.8%) | | 0.65 (0.39–1.08) | | 0.10 | 0.70 (0.41–1.19) | | | 0.180 | |
|  | E | 2/33 (6.1%) | | 0.31 (0.08–1.27) | | 0.10 | 0.35 (0.08–1.46) | | | 0.150 | |
| ^✝^: at enrolment;^🞰^: 3 months pre-enrolment; ^1^ Covariates for the adjusted models in this table include: age less than 25, NG or CT positive at enrollment, any contraceptive use at enrollment, more than one sexual partner at enrollment, education status, marital status at enrollment, last partner HIV status, and any transactional sex in 3 months pre-enrollment, and clinic site | | | | | | | | | | | |
