# Supplementary material for: Oral preexposure prophylaxis use and the risk of bacterial sexually transmitted infections and HIV among African women: A prospective observational cohort study
Source: PLoS Med. 2026 Mar 9;23(3):e1004962. doi: 10.1371/journal.pmed.1004962 (PMC13002101; doi:10.1371/journal.pmed.1004962)
Supplement: S1 File — PrEP-aring family planning clinics to streamline integration of HIV prevention services for young women in Kenya. The Family Planning plus HIV prevention (FP-Plus) Project. (PDF) [file pmed.1004962.s005.pdf]

IMPLEMENTATION PROTOCOL

PrEP-aring family planning clinics to streamline integration of HIV prevention services for young women in Kenya

The Family Planning plus HIV prevention (FP-Plus) Project

Version 3.0

16 February, 2022

Funding:

United States National Institutes of Health

LEADERSHIP TEAM

University of Washington, Seattle, USA

Kenneth Mugwanya, MBChB, MS, PhD (Principal Investigator)
(Responsible for the overall program development, oversight, and science of PrEP components of the program)
Assistant Professor
Departments of Global Health, University of Washington
Harborview Medical Center, 325 Ninth Ave., Box 359927, Seattle, WA 98104
Tel: +1-206-520-3808
Fax: +1-206-520-3831
Email: mugwanya@uw.edu

Jared Baeten, MD, PhD
(Responsible for the overall project development and oversight and science of PrEP components of the project)
Vice Dean, School of Public Health
Professor, Departments of Global Health, Medicine, and Epidemiology, University of Washington
Harborview Medical Center, 325 Ninth Ave., Box 359927, Seattle, WA 98104
Tel: +1-206-520-3808
Fax: +1-206-520-3831
Email: jbaeten@uw.edu

Ruanne Barnabas, MBChB, DPhil
(Responsible for protocol, data and analysis plan, and evaluation of the health economics)
Associate Professor
Departments of Global Health, Allergy and Infectious Diseases, University of Washington
Harborview Medical Center, 325 Ninth Ave., Box 359927, Seattle, WA 98104
Tel: +1-206-520-3813
Fax: +1-206-520-3831
Email: rbarnaba@uw.edu

Deborah Donnell, PhD
(Responsible for protocol, data and analysis plan, and statistical analyses)
Member, Fred Hutchinson Cancer Research Center
Affiliate Professor, Department of Global Health, University of Washington
1100 Fairview Ave, M2-C200, PO Box 19024, Seattle WA, 98109
Tel: +1-206-667-5661
Email: deborah@fredhutch.org

Bryan Weiner, PhD
(Responsible for protocol, implementation science overview)
Professor
Departments of Global Health and Health Services, University of Washington
University of Washington, Box 357965, Seattle, WA
Tel: +1-206-221-7882
Email: bjweiner@uw.edu

Kristin Beima-Sofie, MS, PhD
(Responsible for protocol, develop and refine intervention, Implementation science, M&E lead)
Acting Assistant Professor
Department of Global Health, University of Washington
Harborview Medical Center, 325 Ninth Ave., 359909
Tel: +1-206-520-3869

Email: beimak@uw.edu

Caitlin Scoville, MPH
(Responsible for program coordination, IRB communication, protocol development, data collection, and analysis)
Department of Global Health, University of Washington
Harborview Medical Center, 325 Ninth Ave., Box 359931, Seattle, WA 98104
Tel: +1-206-520-3809
Email: cwscov@uw.edu

Kenyatta National Refferal Hosiptal Nairobi, Kenya

John Kinuthia MBChB, MMed, MPH (Site Principal Investigator)
(Responsible for the development and overall program oversight and science and overseeing all implementing team members in Kenya)
Head, Department of Research & Programs, Kenyatta National Hospital
PO Box 20723-00202, Nairobi, Kenya
Tel: +254 0722 799-052
Email: kinuthia@uw.edu

Daniel Matemo (Site Program Assistant Coordinator)
(Responsible for lab and site protocol development, ERC Communication, data analysis and staff oversight)
Research & Programs,
Kenyatta National Hospital
Tel: 0721230652
Email: dmatemo@uwkenya.org

National AIDS/STD Control Programme, Nairobi, Kenya

Catherine Ngugi, MBChB, MMed (Co-Investigator, NASCOP)
(Responsible for guiding study development and implementation to align with NASCOP activities)
National AIDS and STI Control Program (NASCOP)
Ministry of Health, Nairobi Kenya
P.O. Box 19361-00202
Nairobi, Kenya
Tel: +254 20 2729502
Email: headnascop.moh@gmail.com

Mary Mugambi, MBChB, MPH (Co-Investigator, NASCOP)
(Responsible for guiding study recruitment in collaboration with NASCOP activities)
National AIDS and STI Control Program (NASCOP)
Ministry of Health, Nairobi Kenya
P.O. Box 19361-00202
Nairobi, Kenya
Tel: +254 20 2729502
Email: mugambi49@gmail.com

Kisumu County Government

Dickens Onyango
(Responsible for guiding project development and implementation to align with Kisumu County activities)
Kisumu County Department of Health

P.O. Box 721-40100
Kisumu, Kenya
Email: macdickens2002@gmail.com

COLLABORATING INSTITUTIONS

Kenyatta National Hospital

National AIDS/STD Control Programme

University of Washington

FUNDING AGENCY

Funding Type: Grant
Name of Funding Agency: NIH/NIMH
Principal Investigator of Proposal: Kenneth K. Mugwanya
Proposal Identification Number: 1 R01 MH123267-01
Title of Proposal: Integrating PrEP delivery in family planning clinics in Kenya
Approval Period: 05/01/2020 – 04/31/2025

TABLE OF CONTENTS

COLLABORATING INSTITUTIONS **4**
FUNDING AGENCY **4**
ACRONYMS **6**
SUMMARY **7**
INTRODUCTION/BACKGROUND **9**
LITERATURE REVIEW **9**
PROBLEM STATEMENT **11**
INNOVATION **11**
JUSTIFICATION **11**
IMPLEMENTATION APPROACH **12**
OBJECTIVES **12**
Hypothesis 12
Specific Objectives 12
POPULATION, RECRUITMENT, AND SETTING **13**
Eligibility for program activities (non-research activities): 13
DESIGN AND PROCESS OF INTRODUCING PREP DELIVERY IN FP CLINICS **14**
PROGRAM IMPLEMENTATION PROCEDURES- NON-RESEARCH PROCEDURES **15**
RESEARCH PROCEDURES **19**
DATA **23**
HEALTH ECOMOMICS STUDIES **24**
QUALITY ASSURANCE PROCEDURES **25**
DISSEMINATION **25**
HUMAN SUBJECTS CONSIDERATIONS **25**
Informed consent 25
Risks 27
Benefits 28
Confidentiality 29
Study oversight 29
PROGRAM RECORDS **29**
Data ownership 29
Data release/sharing policy 30
LIMITATIONS **30**
EXPECTED APPLICATION OF RESULTS **30**
BUDGET (TOTAL BUDGET PERIOD) APPENDICES/ATTACHMENTS **31**
BUDGET JUSTIFICATION .... ERROR! BOOKMARK NOT DEFINED.
REFERENCES **32**

ACRONYMS

ART	Antiretroviral therapy
CFIR	Consolidated framework for implementation science research
CME	Continuing medical education
DSMB	Data and safety monitoring board
EC	Ethics committee
FP	Family planning
GBV	Gender based violence
HST	HIV testing services
IPV	Intimate partner violence
IRB	Institutional review board
KNH	Kenyatta National Hospital
MCH	Maternal child health
MOH	Ministry of Health
M&E	Metrics & evaluation
NASCOP	National AIDS and STIs Control Programme
ORIC	Organizational readiness for change
PMTCT	Prevention of mother to child transmission
PrEP	Pre-exposure prophylaxis
RE-AIM	Reach effectiveness adoption implementation maintenance
RR	Relative risk
SE	Standard error
SMS	Short message service
SRH	Sexual reproductive health
STI	Sexually transmitted infection
TA	Technical assistance
UW	University of Washington
WHO	World Health Organization

SUMMARY

Women account for a disproportionate proportion of individuals with new HIV infections in Africa and are a priority population for HIV prevention, including provision of pre-exposure prophylaxis (PrEP). PrEP is a highly effective user-controlled prevention method, with potential to reduce incident HIV infections in Africa if delivered with sufficient coverage among at-risk populations. Anchoring PrEP delivery to care settings that women already trust and access routinely may offer an efficient platform to reach HIV at-risk women, but context-specific evaluations are needed.

In Kenya, a country with joint 3rd largest HIV epidemic, family planning (FP) clinics are highly accessed by sexually active women and offer an opportunity to integrate HIV prevention and sexual and reproductive health services, including PrEP provision and management of sexually transmitted infections (STIs). A recent large clinical trial of contraceptive use and HIV acquisition (ECHO Study) found that HIV risk was alarmingly high for FP clinic attendees. These results have emphasized the need to strengthen integration of HIV prevention and FP services. In our recent pilot study (PrEP Implementation in Young Women and Adolescent program), we pioneered PrEP provision in FP clinics and demonstrated that it was possible to integrate PrEP provision in FP systems with program-dedicated staff. Over 1271 women seeking FP services were screened for HIV risk factors in 8 FP clinics, with 22% PrEP uptake in <12 months. PrEP uptake declined substantially when program-dedicated staff left, demonstrating the need for PrEP delivery models that integrate efficiently with existing staff. Using FP clinics leverages the current health system, staffing and supply chain and could address barriers that women face for PrEP access, including lack of time, cost, and potential stigma of visiting a facility solely for HIV prevention. With more efficient PrEP integration, there is opportunity to improve FP care and optimize women-centered care with a one-stop approach to clinic. In 2017, Kenya was the first African country to launch a national PrEP program and implementation is ongoing at different delivery points. Successful implementation of PrEP interventions in Kenya has the potential to influence the delivery of PrEP in other African settings. With a multidisciplinary team and in collaboration with the Kenya Ministry of Health (MOH) and Kisumu County, we will use a multifaceted implementation strategy to promote sustainable integration of PrEP provision in a combination of HIV prevention package for at-risk young women through FP clinics in Kisumu Kenya.

Design: Prospective, open-label implementation science project to catalyze integration of comprehensive HIV prevention and PrEP care services for adolescent girls and young women accessing routine services at public health facilities with established family planning clinics in Kenya. Streamlined screening for HIV risk and PrEP provision will be promoted in 12 FP clinics in Kisumu County according to national guidelines in a staged fashion (a stepped wedge design). A nested research component will establish a prospective open cohort to provide complementary individual-level clinical and behavior outcomes. Follow-up for research procedures will be for up to 24 months at each implementing clinic.

Population: Heterosexual sexually active HIV uninfected young women of reproductive age. Within the large delivery program, data on up to 25000 women accessing 12 FP clinics will be abstracted to understand delivery and optimize integrated implementation of FP and HIV prevention and PrEP care processes. The open cohort will include up to 900 HIV uninfected women (both women who initiate PrEP and those with HIV risk factors but choose not to initiate PrEP). Up to 200 key delivery informants will participate in qualitative interviews about provider, organizational, and health system-level factors that influence implementation processes.

Objective 1: **Deliver PrEP integrated in public health FP clinics in Kenya and evaluate program reach, effectiveness, adoption, implementation, maintenance, and impact**. We will introduce PrEP delivery into 12 FP clinics in Kisumu area of Kenya – a region with an HIV prevalence of up to 28% among young women – using a stepped-wedge randomized trial design. We will rigorously evaluate implementation effectiveness and impact, using the RE-AIM framework. The multifaceted implementation strategy to integrate PrEP in FP clinics will include: 1) provider training; 2) promotion of HIV testing, partner HIV testing, screening for HIV risk and offer of PrEP; 3) technical assistance and supervision; 4) audit and feedback with continuous improvement to optimize delivery; 5) clinic-specific workflow mapping; and 6) streamlined data and PrEP supply systems. Key outcomes will be proportion of women accessing FP clinics who are screened for

HIV risk (Reach), PrEP uptake, PrEP adherence quantified by tenofovir levels in dried blood spots and women staying HIV free (Effectiveness), proportion of targeted providers who offer PrEP (Adoption), proportion of core components of PrEP delivered per protocol (Implementation), and number of clinics delivering PrEP at 6 and 12 months after the end of intensive technical assistance (Maintenance).

Objective 2: To assess clinic readiness to implement, fidelity, impact on current services, facilitators, and barriers to integrating PrEP delivery in FP clinics in Kenya. We will conduct serial mixed-methods quantitative and qualitative assessments of PrEP implementation in FP settings with policy key informants, providers, and women during baseline and implementation periods. We will use validated tools for Organizational Readiness for Implementing Change (ORIC) and the Consolidated Framework for Implementation Science Research (CFIR; Intervention characteristics, inner and outer settings, process) to assess readiness to implement, fidelity, and contextual enablers and barriers of implementation in FP clinics and how clinics innovate efficient delivery systems. We will use this data iteratively to optimize implementation.

Objective 3: To assess incremental costs, budget impact, and affordability of integrating PrEP delivery in FP clinics in Kenya. We will conduct time and motion studies to optimize delivery efficiency, define the costs, and budget impact, and affordability of PrEP delivery integrated in FP clinics. We will conduct micro-costing, staff interviews, and time and motion studies to estimate the cost and model the budget impact of integrating PrEP provision in FP clinics on annual basis, over a 5-year horizon. Costs will be benchmarked against MOH and Kisumu County budgets, evaluated for scenarios with current and future HIV prevention methods mix. The primary analysis will be from the programmatic perspective, with a secondary analysis from the societal perspective to account for client opportunity and financial costs. H ealth outcomes will include incident HIV cases and disability adjusted life years averted.

Objective 4: Refine and optimize simple data tools that will expand and support delivery of integrated family planning and HIV prevention services at scale and secure buy in from relevant stakeholders to ensure delivery continues to scale up at the national level. As part of the Objective 1 delivery work and refined through information learned in Objectives 2 and 3, we will develop and refine data tools, clinical delivery products, and assessment tools. We will also coordinate with clinic and policy stakeholders to optimize PrEP commodity supply chain management, and staff training regarding PrEP in Kenya. Finally, we will work with Ministry of Health, County government, stakeholders in the Sexual and Reproductive Health space, civil society, clinical providers and community to provide the necessary foundation to further expand integrated delivery of HIV prevention services and PrEP and sexual reproductive services at a wider scale in Kenya.

INTRODUCTION/BACKGROUND

African women face disproportionate risk of HIV. More than 1.7 million persons get newly infected with HIV annually, a majority in sub-Saharan Africa where >600 000 new infections occur each year among young women[1]. Moreover, adolescent girls in Africa have a 3-fold higher HIV prevalence than their male counterparts (UNAIDS, 2013). Social, structural, and economic disparities perpetuate vulnerabilities of women[2]. Financial disparities and intimate partner violence often hinder a woman's ability to negotiate condom use and protect herself from HIV. In stable partnerships, condom use is low and women are often unaware of their partner's HIV status[2-4]. Young women may partner with older men for financial support or based on cultural norms[5]. Lack of money, time, and awareness often make it difficult for women to go to clinic solely for HIV services.

While high protective rate of PrEP resulted in world Health Organization (WHO) and national guidelines recommending for PrEP use as an additional tool for prevention of HIV infection among individuals at high risk of acquiring HIV including adolescents and young women, however these guidelines are rarely implemented in family planning clinics and thus a substantial group of women of reproductive age fails to get these services. Alternative models, such as integrating PrEP into family planning clinic, a department that most women trust and feels comfortable sharing their sexual history, could increase uptake of PrEP. In Kenya, data from a pioneer study on provision of PrEP in FP clinics, demonstrated high acceptance of PrEP services at these reproductive health clinics. However, PrEP delivery substantially declined when program-dedicated staff who were providing PrEP left the facilities thus, highlighting the need of empowering and mentoring facilities on integrating PrEP delivery at the FP clinics. We proposal a one stop shop approach, where women seeking family planning services will as well get PrEP services, all in one room. This strategy will provide an opportunity to improve both FP and PrEP services delivery.

LITERATURE REVIEW

HIV incidence is unacceptably high among African women desiring contraception; integrated delivery of HIV prevention and sexual reproductive health services (SRH) is urgently needed. In HIV high burden settings, many women concerned about avoiding or postponing pregnancy are also at elevated risk for HIV. Modern contraceptive methods protect against unintended pregnancies but, with the exception of condoms, no contraceptive method provides protection against HIV or other sexually transmitted infections (STIs). A recent landmark clinical trial in eastern and southern Africa (the ECHO Study)[6], designed to evaluate the risk of HIV acquisition in HIV-negative women who used three common methods of contraception (depot medroxyprogesterone acetate-intramuscular, the copper intrauterine device or a levonorgestrel implant), found no substantial difference in the risk for acquiring HIV between women in the three study groups. However, incidence of HIV infections among the participants was alarming high ─ an average nearly 4% ─ with higher rates of HIV infection for women under 25 years irrespective of the contraceptive method. These results have rightly spurred important discussions about the urgent need to strengthen integration of SRH services with combination HIV prevention, including provision of pre-exposure prophylaxis (PrEP).

Scale up of efficacious HIV prevention strategies is needed to reduce new infections; PrEP is a highly potent, recommended, and impactful strategy for HIV prevention. Recent data from the ECHO Study have demonstrated that HIV remains a significant personal risk and public health challenge for many women in African countries. Now more than before, there is an impressive collection of highly-effective HIV prevention tools[7], including antiretroviral treatment to reduce infectiousness (or U=U)[8, 9] and PrEP to reduce acquisition in at-risk persons[10, 11]. However, scale up of delivery and access for at-risk populations has been very slow, especially in the African settings. PrEP efficacy against HIV was demonstrated in studies among men who have sex with men (MSM) [11], heterosexual men and women[10], and injection drug users[12], in diverse geographic settings. In 2015, the World Health Organization (WHO) issued guidance recommending PrEP as an additional prevention option for all persons at substantial risk for acquiring HIV[13]. Following the release of WHO guidelines, multiple countries released their own national PrEP guidelines including Kenya (2016) which recommends PrEP for all persons at substantial risk of HIV[14, 15], including for at-risk young women, a priority population in this setting. PrEP as a recommended user-controlled strategy[16-19] has the potential to drive the paradigm shift in preventing HIV acquisition especially for women who may be unable to engage their male partner(s) in HIV prevention.

The World Health Organization (WHO) has issued a 'Call to Action' to integrate sexual and reproductive health and rights with HIV prevention services. Integrating family planning (FP) services with HIV services is a recognized key strategy to meet the 2015 millennium development goals[20], but there is a disconnect between policy and practice at country, regional, and facility level in part due to limited funding[21]. The nearly ~4% HIV

incidence among women in spite of full HIV prevention package offered in ECHO study (PrEP use was low) is unacceptably higher than the 3% country threshold suggested by WHO as an indicator of substantial HIV risk for HIV individuals. In the wake of these results, WHO has called for renewed efforts and commitment to support countries with high HIV incidence rates, to develop plans to provide integrated FP and HIV and STI services[20]. It is time to break the silos (*James Kiarie IAS 2019*). Our proposed work in Kenya, one of the four countries where the ECHO study was conducted, is a direct response to this call and will provide first step robust evidence on how to effectively integrate these services in 'real world' settings.

FP clinics are highly accessed by women in Africa and could offer an efficient platform to reach at-risk women for PrEP provision; implementation science evaluations are needed to sustainably bring implementation to scale. In many settings in Africa FP clinics provide broad coverage for women in their reproductive years. In Kenya, 65% of sexually active unmarried women use a modern contraceptive and a substantial proportion (69%) access it through public health FP settings[22]. Thus, anchoring HIV prevention services including PrEP delivery to care settings that women already trust and access routinely offer a great opportunity to efficiently reach HIV at-risk women. By adding PrEP counselling and dispensing to FP clinics it is possible to expand access to an already established at risk group of women accessing FP services. In the recent PrEP Implementation in Young Women and Adolescent program (funded through PEPFAR DREAMS innovation challenge), we pioneered provision of PrEP in FP clinics in Kisumu Kenya and showed that it was possible to integrate PrEP provision in FP systems with a program-dedicated staff[23]. Over 1271 women seeking FP services were screened for HIV risk factors, with 22% PrEP uptake during 12-months of the study period (Figure 1). In that model, study nurses only performed HIV risk counseling and provision of PrEP but did not participate in delivery of FP services; women first completed other services, including HIV testing, and were then referred to a PrEP-dedicated nurse. PrEP delivery substantially declined when program-dedicated staff who were providing PrEP left, demonstrating the need for PrEP delivery models that integrate efficiently with existing staff and clinic systems[23]. Although there are many demands on existing staff, it is plausible that efficient systems could be built to include promotion of HIV testing, knowledge of partner HIV status, and PrEP provision within FP clinics. With more efficient PrEP integration, there is opportunity to improve FP care and optimize women-centered care with a one-stop approach to clinic. The current proposal draws lessons directly from that pilot work, to test a multifaceted implementation strategy model that aims for a fully integrated, sustainable, institutionalized, and cost- and time-efficient PrEP program in African FP settings.

Figure 1. PrEP uptake in FP clinics in Kisumu *(Mugwanya et al PLOS Med 2019)*

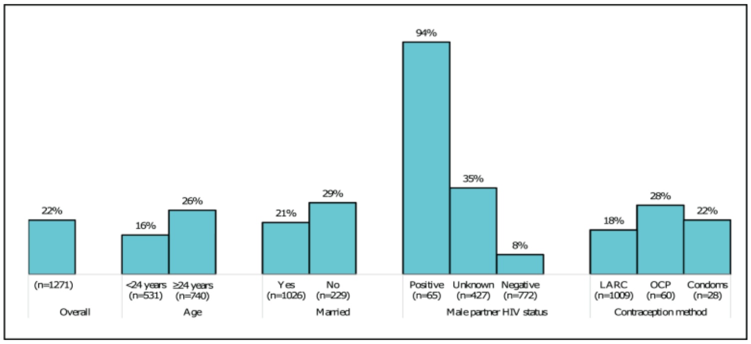

FP-based PrEP delivery could promote women-centered care, expand the reach of PrEP, promote PrEP continuation, and impact: Similar individual, community, and structural determinants of health affect HIV risk and unintended pregnancies, demonstrating how incorporating woman's preferences, goals and context is crucial to service delivery. Using FP clinics leverages current health system, staffing and supply chain and could address barriers that women face for PrEP access, including lack of time, cost, and potential stigma of visiting a facility solely for HIV prevention[24]. Ensuring that young women can access effective contraceptive methods and protect themselves from HIV is critical for women-empowerment and ending the HIV epidemic. There are many advantages to integrate FP and HIV prevention services:

- **The HIV world has a lot to learn from FP programs**: FP has many decades of program experiences, delivery at scale and simplified client-centered care[25]. FP could benefit from academic collaborations and resources from the HIV space.
- **Improved access to both services:** Integrated services can increase access to contraception among clients of HIV services who wish to delay, space, or limit pregnancy, and can also help to ensure safe pregnancy, including primary prevention of mother to child HIV transmission (PMTCT) for those who wish to have a child.
- **Meets women's desires and needs:** Integration permits for one-stop, comprehensive health services where women can receive HIV services at the same place where they access FP services. Women will be more likely to continue in prevention care.
- **Women trust FP providers:** Among >150 U.S. women who reported increased vulnerability to HIV, women

identified FP clinics as places where they saw "trusted providers'[26]. FP providers already counsel women about sexual life and are well-positioned to utilize their experience and skills to implement PrEP for women[27, 28]. The combined need for HIV protection when seeking contraception and the efficiency gained from leveraging existing FP platform, make integration a powerful approach for HIV prevention in young women.

Government support and program ownership is key to success and sustainability of African PrEP programs; Kenya is an ideal setting to test innovations in PrEP delivery. Kenya has the joint 3rd largest HIV epidemic in the world[1], but with wide geographic variation in HIV burden[29]; 65% of all new infections occur in nine out of the country's 47 counties–mainly in the west region (areas around Lake Victoria)[30]. To address this HIV burden, the Kenya Ministry of Health (MOH) developed the HIV Prevention Revolution Road Map: Count Down to 2030, a national plan to drive new HIV infections towards zero, which is based on key concepts in combination prevention: prioritization of sub-populations and delivery of evidence-based combination prevention[29]. In line with this Roadmap, a national PrEP implementation framework was developed by the Kenya MOH[31], with contributions from members of our team[31]. PrEP roll-out nationally in Kenya was officially launched in May 2017, making it the first African national PrEP program and delivery is slowly expanding; ~70% delivery is currently in HIV care clinics[31]. In Kenya's devolved Government structure, the MOH sets and guides national policy but implementation of policy and public health services is under the County Governments. Our proposed work aligns with the Kenya MOH goals, and MOH staff and Kisumu Health leaders will be part of our collaborative team that aim to understand how to integrate and provide comprehensive sexual reproductive and HIV prevention services.

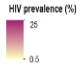

PROBLEM STATEMENT

Similar individual, community, and structural determinants of health affect HIV risk and unintended pregnancies. Despite significant interest in integrating HIV prevention services into sexual, there has been less progress at the global, country, and facility level in practical offer of comprehensive HIV prevention services in FP clinics, including lack of systematic testing services during women visits to, and screening for PrEP eligibility. Specifically, FP-based 'real world' implementation of PrEP delivery has not been explored in an African setting. As PrEP implementation gradually expands in Africa, initial delivery points have centered in HIV care centers focused on serodiscordant couples (70% of PrEP in Kenya is through HIV clinics) and "drop-in" centers and youth-friendly "safe spaces," for key populations. For women, access has been limited mostly through a few demonstration projects in FP and antenatal clinics. The combined need for HIV protection when seeking contraception, widely accessed services, and the efficiency gained from leveraging existing FP platform, make our proposal a powerful and innovate approach for HIV prevention in young women.

INNOVATION

Our proposed project will build on and extend beyond our prior work and leverage the expertise and experience of moving PrEP from the clinical trial to real world delivery. The proposed FP-based PrEP care pathway is novel, sustainable, and translatable to other African settings for a number of reasons. It will leverage existing infrastructure and expertise of FP providers in counseling for sexual history and program delivery on a variety of contraception methods, to embed clinical assessment and PrEP provision in combination HIV prevention package. The proposed work will provide an opportunity for ensuring FP clinics offer an entry point for PMTCT services beyond antenatal care, to prevent primary infection in the first place. Within the standard of care framework, we will promote screening and treatment for STIs, and at subsect of clinics, we will perform baseline etiological testing for curable STIs to document burden, promote partner STI testing and treatment of women infected with STIs, assess incremental costs, and costs that could averted with integrated etiological STI testing. The combined need for HIV protection when seeking contraception, widely accessed services, and the efficiency gained from leveraging existing FP platform, make our proposal a powerful and innovate approach for HIV prevention in young women.

JUSTIFICATION

In Kenya and many other HIV high burden settings, women concerned about avoiding or postponing pregnancy are also at elevated risk for HIV. A recent landmark clinical trial conducted in Kenya and southern Africa to evaluate the risk of HIV acquisition in HIV-negative women who used three common methods of contraception

(depot medroxyprogesterone acetate-intramuscular, the copper intrauterine device or a levonorgestrel implant) (the ECHO Study)[6], found an alarmingly high rates of new HIV infection among all women regardless of contraception method ─ an average nearly 4% ─ with higher rates of HIV infection for women under 25 years irrespective of the contraceptive method. These results have rightly spurred important discussions about the urgent need to strengthen integration of SRH services with combination of HIV prevention, including provision of - PrEP. In Kenya, a substantial proportion of sexually active unmarried women using a modern contraceptive access PrEP through public health FP settings[22]. Thus, anchoring HIV prevention services including PrEP delivery to care settings that women already trust and access routinely offer a great opportunity to efficiently reach HIV at-risk women. In the recent PrEP Implementation in Young Women and Adolescent program (funded through PEPFAR DREAMS innovation challenge), we pioneered provision of PrEP in FP clinics in Kisumu Kenya and showed that it was possible to integrate PrEP provision in FP systems with a program-dedicated staff. In collaboration of the Kisumu County and MOH/NASCOP, we will extend our experience and lessons learned in feasibility study to study fully aiming for full scale up and efficient integration with existing services and sustainability beyond the program.

IMPLEMENTATION APPROACH

This a prospective, open-label implementation project to catalyze integration of HIV prevention and PrEP care services for adolescent girls and young women in family planning clinics in Kenya. We will conduct a stepped-wedge cluster-randomized programmatic project to integrate PrEP provision in combination of HIV prevention package in 12 family planning clinics in Kisumu, Kenya– a region with an HIV prevalence of up to 28% among young women. We will optimize and sustain PrEP delivery with existing FP staff supported through training and ongoing technical assistance. We will rigorously evaluate program reach, effectiveness, adoption, implementation, maintenance, and impact using the RE-AIM framework[32] and how clinics build new efficient delivery systems.

OBJECTIVES

Evaluate the effectiveness of program implementation, barriers, and facilitators to integrating PrEP delivery in family planning clinics

Hypothesis

We hypothesize that sustainable delivery by existing staff will be feasible and integrated FP and HIV prevention services will provide women-centered services with a 'one-stop' location, promote reach for at-risk women, continuation in prevention care, and improve the overall impact of PrEP.

Specific Objectives

Objective 1: Deliver PrEP integrated in public health FP clinics in Kenya and evaluate program reach, effectiveness, adoption, implementation, maintenance, and impact.
Key outcomes: Number and proportion of women screened for HIV risk (Reach), PrEP uptake, Number and proportion of providers trained and delivering PrEP (Adoption), PrEP adherence, HIV infection, Number of women screened and treated for STIs, proportion of women with partner tested for HIV, Number and proportion of women receiving contraception and type of FP method, number of clinics implementing after intensive technical assistance (TA)

Objective 2: To assess clinic readiness to implement, fidelity, impact on current services, facilitators, and barriers to integrating PrEP delivery in FP clinics in Kenya.
Key outcomes: Acceptability, fidelity, facilitators, barriers, and client satisfaction

Objective 3: To assess incremental costs, budget impact, and affordability of integrating PrEP delivery in FP clinics in Kenya.
Key outcomes: Incremental costs, patient opportunity and financial costs (e.g., clinic time), HIV infection averted.

Objective 4: Refine and optimize simple data tools that will expand and support delivery of integrated family planning and HIV prevention services at scale and secure buy in from relevant stakeholders to ensure delivery continues to scale up at the national level.
Key outcomes: FP register and PrEP M & E tool completion rates, indicator reporting rates to MOH/County, commodity stock outs

The project consists of two key distinct but complementary components:

1. **Program delivery procedures:** The program delivery component includes routine procedures for delivery of integrated HIV prevention and PrEP services as part of standard of care (HIV testing and counseling, behavior risk assessment for PrEP eligibility, PrEP and ART prescription, adherence counseling, and program data abstraction for quality improvement). The procedures are minimal risk as they are the usual procedures that would be ordinarily conducted in routine medical clinics that offer PrEP in Kenya. These procedures will be conducted by the usual clinic staff.

2. **Research procedures:** The research component will include procedures that are not part of direct patient care but are important for deeper understanding of the process to integrate HIV prevention and PrEP services in clinical settings. Research procedures will be undertaken: 1) procedures for open cohort individual clinical outcomes, 2) Quantitative surveys with users and providers, 3) Blood draw at subset of visits for objective evaluation of PrEP adherence among FP clients who are PrEP users; and 4) qualitative interviews with users, providers and key informants to identify facilitators and barriers to implementation. All research procedures will be conducted by the project staff and informed written consent will be obtained from participants for these procedures.

POPULATION, RECRUITMENT, AND SETTING

The population will include consecutive women who are sexually active, not known to be living with HIV, of reproductive years accessing routine services at public health facilities with established FP services in the Kisumu area of Kenya, given it is a high HIV burden area (>19%; overall and >28% among women, compared to 4.6% nationally).

Sites

We have identified 12 potential public health facilities to work with (**Table 1**), with variance in clinic size and catchment area to capture the diversity. The goal of the selection process would be to identify clinics that represent the geographic, economic and demographic characteristics of all women accessing FP services and have bandwidth among the clinic staff. Final clinics will be selected through a joint decision process including study investigators and the MOH and Kisumu County officials. For this PrEP roll-out program, where the goal is for real-world delivery of PrEP as part of national PrEP scale-up program,

Table 1. Potential study site in Kisumu County, Kenya

Health FP facilities	Average monthly number of women attendees	
	New visits	Revisits
Jaramogi Oginga Referral Hospital	351	97
Chulaimbo County Hospital	70	102
Airport Health Centre (Kisumu)	59	70
Muhoroni Sub-county Hospital	34	57
Lumumba County Hospital	177	89
Kombewa County Hospital	62	75
Kisumu County Hospital	147	126
Ahero County Hospital	100	94
Nyakach County Hospital	58	60
Rabuor Sub-County	64	80
Migosi County Hospital	155	57
Manyuanda Sub County Hospital	71	47

Recruitment

Formal recruitment will reflect approaches used by FP clinics in Kenya. In partnership with Kenya Ministry of Health and Kisumu County program we will establish and promote HIV screening methods for HIV risk that operationalize protocol-specified requirements for eligibility determination in a manner that is tailored and efficient for integration of PrEP delivery in FP settings in Kisumu Kenya. Strategies will reflect those that are commonly used by public health systems but streamlined to educate women about the importance of HIV testing, knowledge of male partner HIV status, risk factors for HIV of transmission and sexually transmitted infections, and the benefits of ART for treatment of HIV- positive persons and prevention of transmission, and PrEP for HIV-negative at risk for HIV.

Eligibility for program activities (non-research activities):

For this implementation science study, the program will be set up for all women of reproductive age, receiving routine services at the implementing facilities using FP clinics as the delivery point for PrEP. Broad eligibility criteria will reflect the public health nature of this work – to operate effectively, efficiently, and ethically in community settings, PrEP will need to be broadly accessible to all at-risk adolescent girls and young women who shoulder the biggest burden of HIV infections in this setting.

For women

- Of reproductive age, ≥15 years
- Accessing services at implementing clinics.
- Not known to be HIV infected at the time of seeking services
- Has otherwise no other reason which prevent them from participating in the program

For key delivery informants (providers and policy makers)

- Able and willing to provide consent

DESIGN AND PROCESS OF INTRODUCING PREP DELIVERY IN FP CLINICS

Systematic integrated screening for HIV risk and counseling for and provision of PrEP will be introduced and actively promoted in family planning clinics in Kisumu. These strategies will be introduced in staged three successive waves (steps), occurring every 3 months, with 4 clinics per step or stepped-wedge design (**Figure 2**). The order at which clinics initiate the implementation will be determined by randomization stratified by clinic size, identified as likely to have a marked effect on implementation outcome. Randomization process will take place at a meeting with the Kisumu County Health leadership, implementing health facilities and led by the project staff. Prior to initiating implementation of the program, we will conduct clinic-wide training for health providers at each clinic and we will engage existing FP providers in continuous quality improvement to optimize delivery. At all implementing clinics, we will conduct a pre-implementation needs assessment and observation of current services to optimize data and PrEP supply systems. During this pre-implementation period, a minimum of 3 months for each clinic, we will assess and document current processes on HIV counseling and testing, male partner testing, PrEP counseling and use (expected to be minimal), STI counseling and treatment, male partner STI testing and treatment of women infected with STIs, incident HIV and STI. During implementation, we will optimize delivery of PrEP in combination HIV prevention using existing FP clinic staff supported through ongoing technical assistance from program technical team and MOH/NASCOP and Kisumu County Health supervision team to audit for process indicators of implementation progress and continuation quality improvement. On-the-job training of new staff will be promoted and we will conduct refresher CME-like training quarterly or as needed.

Figure 2. Schematic of stepped wedge cluster randomized trial. Outcome assessment begins at baseline. 4 clinics initiate the intervention at every each 3-month step

Clinics Randomly assigned	Baseline	Step 1	Step 2	Step 3	Step 4	Step 5	Step 6	Step 7	Step 8	Step 9	Step 10
Group 1 (4 clinics)	S	I	I	I	I	M	M	M	M	M	M
Group 2 (4 clinics)	S	S	I	I	I	I	M	M	M	M	M
Group 3 (4 clinics)	S	S	S	I	I	I	I	M	M	M	M

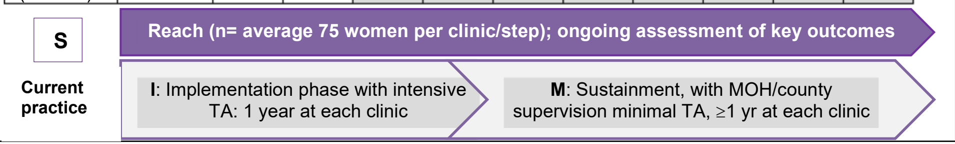

Sample size and power: For this facility-level intervention, the co-primary outcomes will be: 1) proportion of women accessing FP services that are counseled and screened for HIV risk, and 2) proportion of women who initiate PrEP. HIV testing is the entry point for all HIV services. Although HIV counseling and testing an expected service in FP clinics, it is not routinely done in most FP settings. Assuming a conservative 50% rate in the current practice, with 3600 total women screened, we will have ≥80% power to detect a meaningful increase of 10% or more in proportion of new clients screened for HIV prevention. We assume the average proportion being screened at project start will be between 50% and 70%[33, 34]; accounting for heterogeneity in baseline levels and treatment effect, we will have ≥80% for the range of likely baseline levels of screening. We expect that prior to starting the intervention, PrEP initiation will be very low, averaging <2% (ranging from 0-5% across clinics). After the intervention, we expect to see PrEP initiation among general population women increase to a mean of 22% (based on preliminary work, Mugwanya et al *PLOS Med 2019*), much higher among women with risk factors for HIV. Sample size and power calculations for stepped wedge cluster randomized trials should account for possible heterogeneity in intervention effects so that the study is not underpowered[35]. We expect that the overall increase of +20% will reflect a range of true intervention effects across clinics depending on clinic factors, likely distributed between 10%-30%. Accounting for heterogeneity in baseline levels and intervention effect, if each clinic offers universal counseling for HIV risk and offer of PrEP for at least 100 new women per step, totaling at least 4800 new women overall, using a two-sided α=0.05, the study will have excellent power to detect the expected increase in PrEP initiation (**Table 2**). To fully understand how public health clinics build new efficient systems for delivery of integrated FP and HIV prevention services, we will abstract data on all HIV-uninfected women accessingservices in implementing clinics in order to establish the underlying denominator in the program. Based on data from the baseline preparatory periods (monthly volume of ~1000) and a conservative PrEP screening rate between 10-30%, we estimate that program data on up to 25000 women accessing services across the 12 FP clinics will be needed for thorough evaluation of this new program beyond the statistical sample size. This sample size will permit a full-scale test of the system, since the efficiency in delivery is in large part at the system level, above and beyond the individual client encounter.

Table 2: Power to detect an increase of 10-15% in counseled and screened for HIV risk in stepped wedge design with 12 clinics and 3 steps.

Total clients in study	Effect size	% new clients screened for HIV prevention		
		Current practice	Intervention	Power (%)
**3,600**	10%	50%	60%	83.8%
		60%	70%	86.2%
		70%	80%	90.8%
	15%	50%	65%	98.8%
		60%	75%	99.2%
		70%	85%	99.7%

Power to detect an increase from baseline of 5% to 20% in PrEP initiation using the stepped wedge design with 12 clinics and 3 steps.

Mean clients per clinic, per step	Total clients per step	Number screened	Power (%) at varying effect sizes			
			Effect size, increase in PrEP uptake			
			+5%	***+7.5%***	***+10%***	***+20%***
**100**	**1200**	**4,800**	**84%**	**>99%**	**>99%**	**>99%**
75	900	3,600	80%	98%	>99%	>99%
50	600	2,400	73%	95%	99%	95%

PROGRAM IMPLEMENTATION PROCEDURES- NON-RESEARCH PROCEDURES

Program non-research activities will include routine care services which are part of standard services for administration of HIV prevention services including PrEP provision. At each clinic, staff delivering HIV prevention including dispensing PrEP will be the usual clinic staff (clinicians, nurses, counselors). The program delivery component includes routine procedures for delivery of integrated PrEP and SRH services as part of standard of care (HIV testing and counseling, behavior risk assessment for PrEP eligibility, PrEP prescription, adherence counseling, and program data abstraction for quality improvement). These procedures are minimal risk as they are the usual procedures that would be performed during routine medical care in these clinics or other clinics offering PrEP in Kenya.

Key strategies to integrate HIV prevention and PrEP services in FP clinics (detailed in Table 3). For this implementation program, multifaceted implementation strategies to integrate and promote streamlined HIV prevention services and PrEP provision in FP clinics will include:

Clinic readiness to implement PrEP in FP clinics: Organizational readiness to implement change, the extent to which organizational members are psychologically and behaviorally prepared to implement organizational change[36-40], is an important driver of effective implementation of new public health programs. We will use Dr. Bryan Weiner's theory of organizational readiness for change (ORIC)[41] to define facets of

Table 3. Planned strategies to integrate HIV prevention services and PrEP provision in FP clinics in Kenya

Domain	Provider training	Technical assistance & supervision	Audit and feedback	Clinic flow mapping	Streamlined data and PrEP supply systems.
**Actor**	Project TA	Project TA, MOH and County health supervisors	TA, MOH & County health supervisors	Project staff/nurses	Project staff/nurses
**Action**	Training on HIV prevention and PrEP services and MOH M & E tools	Coach and mentor clinics/providers on PrEP delivery	Program data abstraction, clinic-level reports for continuous quality improvement	Client and workflow mapping to identify bottleneck and opportunity for efficiency	Streamline PrEP commodity supply to FP clinics. Optimize simple data systems and data utilization
**Target of action**	Health providers in FP clinics	Health providers in FP clinics	Clinic manager/ Health providers in FP clinics	FP clinics/clinic staff	FP clinic systems/Clinic managers
**Temporality**	2-4 week prior to implementation	Baseline	Implementation	Baseline	Baseline
**Frequency**	MOH PrEP curriculum delivered in 5 modules over 2 weeks at the facility premises. Ongoing refresher 3-6 months or as needed	Weekly TA visits for 6 months, then monthly for 6 months. 3 monthly with study nurse for 18 months then 3 monthly alone for 6 months.	2 weekly for 3 months, then monthly for 9 months	At baseline, 6, and 12 months	Baseline, then ongoing and fully optimized within 12 months.
**Outcomes**	# of providers trained, provider knowledge gain, confidence to deliver PrEP	Fidelity, Maintenance (sustainability)		Adaptations to clinic flow. Client time in clinic, provider time spent to provide services. PrEP continuation	Data and M & E tools completeness PrEP commodity stock out

readiness (i.e., change commitment and change efficacy), their immediate determinants, and how they impact implementation. Change commitment will reflect clinic staffs' shared resolve to implement PrEP in FP clinics and change efficacy will reflect clinic staffs' shared belief in their collective capability to implement PrEP in FP clinics. At each clinic prior to implementation, we will administer a validated ORIC psychometric tool[42] to clinic managers and providers to assess for determinants of readiness to implement, including provider knowledge and confidence to provide PrEP, perception of resource availability, and situational factors including timing of the program. We will use this data to understand clinic-level variations in implementation performance, both high and low performing clinics, and how they affect implementation outcomes. If a FP clinic reports too little readiness, the project team will assess the situation with FP clinic leaders and county health team, determine if a brief intervention to address readiness problems could be performed to raise the level of readiness prior to implementation initiation, and if so then take those actions (e.g., raise staff motivation via championing / persuasive communication from clinic directly, do a brief educational session to address staff attitudes/perceptions of sexually active women, have clinic leaders and county team assure staff that client flow and workflow mapping will identify opportunities to incorporate delivery into routine work, etc.). If the assessment indicates that the readiness problems cannot be addressed briefly or easily, then the "not-ready" FP clinic would be replaced with a "more ready" one prior to implementation initiation.

Implementation of HIV prevention and PrEP delivery services integrated with FP services: We will optimize PrEP delivery in FP clinics using existing FP clinic providers. For this real-world PrEP implementation program, all eligible women accessing routine services at the implementing facilities and interested in PrEP will have an opportunity to receive PrEP services through FP clinics regardless of their initial purpose for visiting the facilities. The goal is to make FP clinics the focal point for women-centered HIV prevention and PrEP care. For women not primarily accessing FP services but who receive their PrEP care through FP clinics, only their HIV prevention/ PrEP-related records from program standard HIV prevention tools, including RAST tool, PrEP card, MOH registers will be used for program evaluation and improvement. The core components of PrEP delivery– including screening for HIV risk, HIV testing, dispensing, adherence and risk reduction counseling, assessment of side effects, and provision of refills, or laboratory safety assessment for creatinine – will be conducted by existing FP clinic staff as part of standard care service package – the goal is to catalyze sustainable scale-up within existing structures beyond the study). Within the standard of care framework, clinics will promote a comprehensive provision of integrated FP services and HIV prevention services, including contraception methods uses and preferences, PrEP provision, counseling for HIV risk and testing, promotion of knowledge of partner HIV status, condom provision, and screening and treatment of STIs. Counseling, PrEP eligibility determination, clinical provision of PrEP and follow-up will follow the Kenya national PrEP guidelines using MOH/NASCOP risk assessment screening

tool and clinical encounter form[43]. Specifically, consecutive women will be counseled for HIV risk and those eligible or interested in PrEP will be offered same day initiation. Current MOH/NASCOP PrEP guidelines recommend visits after PrEP initiation at month 1 and then 3 monthly with re-evaluation of ongoing HIV risk, sides effects, adherence and indication for PrEP.

PrEP medications: HIV uninfected women at substantial risk for HIV infection who choose to initiate PrEP and are medically eligible will receive PrEP as part of the Kenya national PrEP scale-up program. PrEP medication and dosing will follow the Kenya guidelines on Use of Antiretroviral Drugs for Treating and Preventing HIV Infections in Kenya. PrEP medications and commodities will be provided by the Kenya national stock of antiretroviral as part of the national PrEP scale-up program.

Support for continuation and adherence to PrEP: Adherence is a key driver of PrEP effectiveness. For this PrEP roll-out program, where the goal is for real-world delivery of PrEP as part of national PrEP scale-up program, formal support for continuation and adherence will reflect approaches used by FP as well MCH clinics in Kenya. Women using PrEP will receive ongoing adherence counseling and support as part of program services. We think that women who choose to initiate PrEP as their preferred HIV prevention option will be more motivated to adhere to PrEP and that PrEP delivery models that systematically assess risk and optimize partner HIV status ascertainment will enhance adherence among at-risk women. Mobile phone-reminder systems are effective and recommended approach to support adherence to ART in HIV-infected persons and are routinely used by some clinics in Kenya to support routine care services including engagement with MCH and ART adherence in PMTCT. Promotion of adherence to PrEP will be done as part of standard of care services in the clinic and will include extending experiences learned from the ART program and recently from PrEP program. Women who initiate PrEP and feel comfortable about adherence support communication on their phone, will have an opportunity to receive brief mobile-phone communication to augment their adherence to PrEP and to understand individual-level HIV prevention behavior.

Promotion of male partner testing: As part of efforts to promote integration of comprehensive HIV prevention services for at-risk women, we will work with clinics to enhance the existing systems in FP clinics to encourage male partner testing. Strategies to promote knowledge of male partner status may include extending invitation to male partners to come to clinic for HIV testing or distribution of self-test kits to deliver to male partners to self-test. Promotion of male partner testing will be done as part of standard of care services with strict observance of ethical considerations, including consideration for potential partner violence or social harm. Specifically, providers will be trained to screen for women who believe violence could occur when they try to distribute HIV self-test kits or invite their partner for testing; these women will not be offered HIV test kits but will receive all other HIV services including PrEP. Women who are comfortable delivering HIV self-test kits or inviting their sexual partner(s) for testing, will be counseled and trained on ways to determine whether to discuss HIV testing or offer a self-test kit to their sexual partner(s). Follow-up information about the partner testing including on HIVST will be obtained from women by self-report using a brief standardized tool at their routine follow-up visits at all clinics. <u>Women may also consent orally to have this information proactively obtained using SMS or phone.</u> Follow-up information will inquire whether the partner received invitation or the self-test kits, used the kits, test results, and if there was any harm or negative reactions from the partner regarding the test. The active follow-up will be conducted using automated SMS messages sent by a third party (e.g., mSurvey). The women will not be charged for the responses. If a woman does not respond to the SMS, a nurse will call the participant to ask the same questions.

FP services: Based on integrated delivery framework, FP and HIV preventions services will be delivered by the existing FP staff in the same place. We will evaluate the impact of added services on current FP services.

<u>Training and capacity building for PrEP implementation</u>: Training health care providers is an essential component of integrating a new clinical strategy in public health settings. The program will work with MOH/NASCOP and Kisumu County Health Team to support clinics' readiness to deliver PrEP in a combination HIV prevention package in FP clinics. Training will be conducted at each of the participating clinics (in-facility training) using the approved case-based interactive Kenya MOH PrEP training curriculum[31]; our team contributed to the development of this curriculum. Content specific to FP clinics will

be added, including topics on FP, STI, treatment and partner testing services with the goal of equipping providers with the comprehensive knowledge and skills to provide integrated FP and combination HIV prevention services including PrEP services. A standardized pre-and post-test about PrEP will be used to assess knowledge gain. The training will be conducted at each facility premises and curriculum modules will be delivered over a 2 weeks period immediately prior to implementation, and then CME-like quarterly refresher training during delivery. Clinics will begin PrEP delivery after the training. For each training, as part of quality improvement processes, we will administer a standardized pre- and post-test. All trainees will receive their pre- and post-test scores. We will also summarize de-identified scores of the trainees by cadre to be shared with MOH/NASCOP and Kisumu County Health Team, in order to generate discussions that will foster quality improvement processes.

PrEP technical assistance and county supervision: We will conduct ongoing technical assistance to clinics using trained program nurses to coach, mentor, and guide FP clinics to troubleshoot emerging challenges with integrating new services. Technical advisors (TA) will be project nurses with training in PrEP delivery. Prior to implementation, Program nurses will undergo an intensive 2 weeks didactic and protocol training conducted by study investigators, in collaboration with Kenya MOH and Kisumu Health officials. TA will conduct structured periodic visits to each clinic to coach and mentor the health care providers and observe and document PrEP implementation processes with a specific focus on processes that are effective or not and aspects of delivery that are changing with time. Using rapid-cycle analysis approaches, TAs will make summary reports at the end of each clinic visit highlighting what changed, why it changed, who initiated the changes, and outcome of any changes instituted. Best practices will be shared across all clinics for possible adoption. For the first 3 months post-introduction, study nurses (TAs) will visit clinics weekly for 3 months, and then complete monthly visits through the 12-month intensive implementation phase. During year 2, TA activities will scale back to observe sustainment phase. Throughout the first 18 months, the TAs will be accompanied to facility visits by County health and or Kenya MOH/NASCOP supervisors to audit for process indictors for FP services, HIV prevention services, and PrEP and progress.

Novel clinic and patient flow mapping: We will conduct workflow mapping at all sites to identify potential bottlenecks, differences and commonalities among sites at baseline and 6 monthly. We will solicit provider engagement in defining new flow maps to accommodate new tasks and patient flows. Current and future flow maps will be created for each site to demonstrate new pathways.

Audit and feedback and continuous quality improvement: We will conduct monthly audit and feedback activities to track progress and promote continuous quality improvement using clinic-context data reports. Key emerging themes that impact delivery processes will be quickly fed back to the facility for possible actions. Best practices for demand creation, clinical PrEP provision, male partner engagement and HIV testing, clinic flow, and retention strategies will be discussed with and shared across clinics for possible adoption. Detailed clinic-level reports will be generated at baseline and 6-monthly to triangulate delivery processes including adherence to or adaptations to core components of PrEP delivery and impact on FP services.

Streamline clinic data, program data abstraction, and PrEP supply chain: Routine program data are riddled with incompleteness, which may affect effective evaluation of public health programs. During the pre-implementation, we will work with clinics and Kisumu Health leadership to get PrEP commodities (e.g., PrEP, HIV tests) to FP clinics, develop accountability and tracking systems. We will work with clinics and MOH/NASCOP to define and optimize simple sustainable data systems that can utilize clinic-level data and reports to improve services. This may include modification of current PrEP clinical encounter form as well as FP registers or electronic medical records (existing in a few clinics but generally not in FP clinics) to include indicators for STI counseling and treatment, PrEP use, and tracking. As part of the process to optimize data systems and quality improvement, program nurses will abstract some routine program data from (e.g., risk assessment tool or RAST, PrEP clinical encounter/PrEP registers, FP registers) to evaluate how effectively and efficiently integrated delivery of FP and comprehensive HIV services including PrEP services is disseminated and implemented. With the introduction of PrEP as part of routine HIV prevention services, indictors of current standard HIV prevention care, male partner testing, PrEP use (expected to be minimal) have been added to list of indictors to the Ministry of Health. We will work with clinics to promote reporting of HIV prevention service and PrEP indicators to the County and MOH/NASCOP information

systems. We will optimize the data systems with a dedicated data clerk embedded in each of the clinic starting in the pre-implementation period and transition to clinic staff after 12 months.

Cross-sectional and Program exit surveys: User experience is an important part of a successful program service delivery. Within the program we will use cross-sectional quantitative and exit surveys administered to random women at clinic to understand quality of care services and women's satisfaction with services. Exit surveys will be conducted within 3 months of program start and then 6 monthly by trained staff, as clients are exiting the facility. The cross-sectional quantitative surveys will help obtain important data that cannot be obtained from program tools (e.g., the FP register) including detailed demographics and HIV risk behavior characteristics of women accessing FP services. Exit surveys will inform program continuous quality improvement activities.

RESEARCH PROCEDURES

The project will have a nested research component with procedures that are not part of direct patient care but are important for deeper understanding of the process to integrate HIV prevention services broadly, including PrEP services in clinical settings. Informed consent will be obtained for all research procedcures not related to routine care before any procedure is conducted (more details in Human subject section). Research procedures will include:1) an open cohort to assess individual level outcomes; 2) Blood draw for objective assessment of adherence to PrEP for women who initiate PrEP; 3) Qualitative interviews with women, providers and policy key informants; 4) quantitative questionnaires with users, providers and policy key informants; 5) Phone/SMS support for PrEP adherence and or partner testing follow up outcomes and prevention behavior.

Open cohort for individual outcomes*:* Within the programmatic setup, we will establish an open cohort of HIV-uninfected women to closely measure individual-level clinical outcomes on HIV and sexually transmitted infection, PrEP use, and adherence. The cohort will enroll up to 900 HIV uninfected women overall (both women who initiate PrEP and those with HIV risk factors but choose not to initiate PrEP). The cohort will include sexually active women identified with any behavioral factor defined by the Kenya PrEP guidelines to indicate a substantial ongoing risk of acquiring HIV.

Eligibility for open cohort:

- Of reproductive age, ≥15 years
- Sexually active
- Able and willing to provide consent for follow-up in the cohort
- HIV uninfected at baseline based on negative HIV tests, per Kenya national guidelines
- Has at least one risk factor which puts them at substantial on-going risk for HIV. The factors defined by NASCOP include: a) Inconsistent or condom use; b) having a sex partner(s) high risk & HIV status is unknown; c) engaging in transactional sex; d) Ongoing Intimate Partner Violence (IPV) and Gender-based Violence (GBV); e) Recent bacterial sexually transmitted infection; f) Recurrent use of post-exposure prophylaxis; g) Recurrent sex under influence of alcohol/recreational drugs; h) Injection drug use with shared needles and/or syringes; and i) In HIV serodiscordant partnership where the HIV-infected partner has either not initiated ART or is not virally suppressed or a couple wants to conceive.

Procedures and follow-up for the open cohort (Table 4):

Women enrolled in the research open cohort will have research visits at enrollment, one month and then quarterly for up to 24 months with HIV testing, promotion of male partner HIV/STIs testing (e.g., partner invitation or distribution of self-test), and a brief questionnaire on demographics, mental health, sexual behaviors, HIV and STI risk, FP and prevention methods use. Vaginal swab or urine for STI testing and blood sample for plasma or dried blood spot will be archived from women in the cohort, for testing including tenofovir levels for women using PrEP as an objective measure of PrEP adherence. HIV testing will be performed in line with national HIV testing algorithms for Kenya. All counseling and testing approaches will be in accordance with national HIV counseling and testing guidelines. For initially HIV uninfected participants who seroconvert, a plasma sample or dried blood spots will be collected and archived for tenofovir levels and resistance testing. All research procedures for the cohort activities will be conducted by study staff. Written informed consent will be obtained for all research procedures not related to routine care. Consenting process will emphazie to women that participation or non-participation in the research component will not in anyway affect there usual care, including access to PrEP. Participants will receive compensation for their time and transportation expenses to participate in the study procedures at follow up visits.

Table 4. Schedule of procedures for the open cohort

	**Screening/Enrolment**	**1**	**3**	**6**	**9**	**12**	**15**	**18**	**21**	**24**
**Schedule of procedures**										X
Consent for the study	X									
HIV risk counselling	X	X	X	X	X	X	X	X	X	X
Rapid HIV testing	X	X	X	X	X	X	X	X	X	X
Urine and blood sample for plasma and DBS (TDF Levels for those taking PrEP) and archiving	X	X	X	X	X	X	X	X	X	X
Promotion of male partner testing (Invitation or distribution of HIVST)	X	X	X	X	X	X	X	X	X	X
Brief questionnaire (mental health, sexual behavior & HIV risk, medical, & FP history)	X	X	X	X	X	X	X	X	X	X
Vaginal swab or urine for STI testing	X	[X]	[X]	[X]	[X]	[X]	[X]	[X]	[X]	[X]
Screening for STI & treatment	X	[X]	[X]	[X]	[X]	[X]	[X]	[X]	[X]	[X]

Key clinical outcomes: HIV and STI infection diagnoses, PrEP use, continuation, and adherence. Cohort follow up schedules will be aligned with visits for routine follow up care.

Urine and blood drug levels to evaluate PrEP adherence*:* Adherence is a key driver of PrEP effectiveness and a key indicator for program success[44, 45]. At a subset of visits (randomly selected), urine and blood sample for plasma and or dried blood spot will be archived from HIV uninfected women taking PrEP or who seroconvert, for testing including tenofovir levels (as an objective measure of PrEP adherence). Testing for tenofovir levels using plasma and DBS will be performed at Anderson laboratory at the University of Colorado and urine samples will be tested for the same at University of California San Francisco in the USA. Currently, tenofovir testing is only available in the US, however, it is expected to be available in other countries during the project. Shipped samples will be stored at -80 ^{0}C until the testing is done.

Evaluation of implementation processes, including facilitators and barrier to implementation

Sustainable introduction of new evidence-based interventions into clinical settings is a complex and multifaceted process. We will conduct complementary quantitative and qualitative evaluations[46] to rigorously assess clinic readiness to implement and implementation process factors (the what and how of implementation) including enablers and barriers that affect both process and implementation outcomes (i.e., reach, effectiveness, adoption, intervention fidelity, sustainability) at the policy, facility, provider, and client level. We will use multiple data sources to triangulate data and permit deep understanding of implementation processes. To capture the dynamic changes that occur in integrating new intervention in usual practices, we will make serial assessments of implementation process through program period. Specifically, assessment (qualitative interviews and quantitative surveys and TA summary reports, see details below) at each clinic will be conducted during pre-implementation period and then at least 6 monthly during implementation and sustainment phases of the project.

Qualitative interviews: To gain a deeper understanding of delivery processes including the factors and the extent to which the factors influence access to and use of HIV prevention services including PrEP in FP clinics and at the policy, facility, provider, and client level. Qualitative work will extend the work we have already done in this population, and we will adapt our existing questionnaires and topic guides to focus principally on questions related to delivery of the HIV prevention services and PrEP care in FP clinics (Table 5).

Table 5. Qualitative interview topics and CFIR domains covered

Target	Topics	CFIR domain covered
**Client**	Acceptance, unique needs and experiences, confidence in health system and in PrEP delivery in FP settings. Facilitators and/or barriers/concerns (side effects, clinic time, personal costs, stigma)	Intervention characteristics (relative advantage)
**Provider**	Acceptance, preference, workload, opportunity costs, self-confidence, facilitators, barriers	Intervention characteristics, Individual characteristics, Inner settings, and Process
**Clinic-managers**	Opportunity cost, work and patient flow, impact on current services	Inner settings
**Policy key informants**	Acceptance, readiness to adopt and sustain	Outer settings

Qualitative interviews with women: We will conduct qualitative interviews with up to 200 women (average of 16 per clinic) to assess barriers and facilitators to access to and use of HIV prevention services including PrEP in FP clinics. Qualitative data collection will include serial in-depth interviews using pre-piloted semi-structured guides. Because our aim is for a sustainable HIV prevention program broadly, not specific to PrEP, stratified purposive sampling will be used to ensure that the overall sample includes relevant sub-populations of women including:[47, 48]: 1) women who have risk factors for HIV but choose not to initiate 2) women who choose to initiate PrEP, and 3) women who initiate PrEP but later discontinue to understand motivation and reason for discontinuation. Interviews will focus on understanding women's acceptability, unique needs for HIV prevention and sexual reproductive health, barriers, experiences with integrated delivery services, partner testing, waiting times, confidence in the health system, and stigma related to accessing HIV prevention services broadly, and PrEP in FP clinics. Additionally, we will interview individual women who stopped coming to the clinic for PrEP refills.

Qualitative interviews with health providers, clinic leadership, and policy key informants. Assessing implementation barriers and facilitators for integrating PrEP services in FP clinics throughout the course of implementation can improve delivery and inform future scale-up. In addition to PrEP user-specific interviews, we will study operational delivery, at the level of providers, organization (i.e., clinic), and health system (supply chain, Ministry of Health, county government, other parts of government). Using methods as described below, we will conduct serial interviews with providers at the FP clinic (clinic managers, counselors, nurses, doctors) to understand acceptance, barriers and facilitators, knowledge and confidence in delivering PrEP, and suggestions for efficient delivery in the context of FP clinics. In addition to frontline provider-specific outcomes, we will study health system operational execution at the policy level to explore readiness to adopt and sustain implementation. We will conduct key informant interviews with policy-level key informants at national level (MOH) and County level, targeting key informants from both HIV prevention and sexual reproductive programs. We will also assess for perception on impact addition of PrEP delivery on current FP services (opportunity costs). Interviews will be conducted during baseline (pre-implementation), implementation and the sustainment phase to capture changes, how clinics innovate strategies to improve delivery of integrated services and to assess determinants of successful implementation. We have estimated up to 200 participants will permit us to capture diversity in geography, employment role, and opinion.

Approach for qualitative interviews: As previously stated, we will use the Consolidated Framework for Implementation Science Research (CFIR)[49] to guide development of interview data collection instruments, data analysis, and reporting of findings. Our team has vast experience conducting qualitative and process work to evaluate intervention implementation (Drs. Beima-Sofie, Weiner, Baeten)[50-53], including use of the CFIR to understand determinants of implementation success. This project will adapt existing questionnaires and topic guides to focus on questions related to PrEP delivery in the context of FP clinics. Interviews will be carried out by trained Kenya research assistants with social science expertise. We anticipate that each interview will last 30-60 minutes and be conducted in the participant's preferred language (English or local language). Provider and policy-maker interviews will be conducted at the clinic, or another location of the participant's choice. Interviews will follow semi-structured topic guides, developed through the CFIR website

and adapted based on our ongoing research studies, with flexibility to explore alternative constructs and relevant content. These will provide contextual information on factors important for the intervention's acceptability, adoption, and maintenance, as well as quality and fidelity in implementation. Topic guides for interviews with women will explore personal experiences with accessing and using FP, HIV prevention, and PrEP, and will be primarily based on literature reviews and our ongoing research. All interviews will be recorded with permission, transcribed, and translated into English.

Quantitative questionnaires*:* To complement qualitative interviews*,* we will use brief quantitative surveys with clinic-managers and frontline providers at 12 clinics to gain deep understanding of clinic-level factors that impact implementation, including assessment of quality of care and job satisfaction. These data will provide further insights into low and high performing clinics.

Assessment of fidelity to implementation of streamlined HIV prevention services in FP clinics: We will prospectively document the extent to which the core components the implementation strategy are implemented as planned. We will examine fidelity across dimensions of adherence, exposure, quality of delivery, provider responsiveness, and program differentiation. We will assess and document factors that affect fidelity, including characteristics of providers, clinics, women, the FP or community context, and program support systems (e.g., County supervision, technical assistance). Specifically, we will use unannounced standardized patient actors to measure fidelity of the implementation of comprehensive HIV prevention services including PrEP delivery intervention (i.e., counseling and screening for HIV risk factors, HIV testing, PrEP provision). Rationale*:* Standardized patient actors have been shown to accurately measure care and assess provider performance in a variety of settings.[62–64,121–124] The use of standardized patient actors is advantageous in this project because it enables us to identify weak points in our FP-based PrEP delivery care pathway for women and scenarios that are of particular interest (e.g., which HIV prevention option best suites their situations, or individuals who test HIV-1 positive). Case scripts*:* Study team members will develop case scripts in consultation with PrEP providers. Actors*:* We will hire and train 2-3 professional actors on the case scripts that detail key elements of screening and counseling about HIV risk and provision of HIV prevention, including general PrEP inquires and provision, eligibility requirements, symptoms of PrEP side effects, and PrEP adherence. Unannounced visits*:* The patient actors will visit a subset each of clinics: once to initiate PrEP, and a second time to refill their PrEP drug supply. The patient actors will complete all steps in the based FP-PrEP delivery care pathway that would be completed by any other PrEP client. Standardized patient checklist*:* At the end of each unannounced visit, the standardized patient will complete two checklists: (1) a technical checklist that identifies what services they were offered and (2) a checklist that assess the quality of services received, including duration of the visit and the actors' attitudes on how they were treated by providers. The technical checklists will vary slightly for PrEP initiation (enrollment) and PrEP refill (follow-up) to account for the different services associated with these visits. Data analysis. We will report the percentage of participants who completed receiving the items on the technical and quality-of-care checklists. We will pre-determine what items on the checklist are the key essentials for family planning clinic-based PrEP delivery and calculate the percentage of participants who received all these services. Additionally, we will identify key areas of care pathway that will require additional training, modification, or reinforcement during implementation. As detailed **Table 6**, we will use multiple data sources, including program data, checklists, visit logs, and providers surveys. We will document how clinics make adaptations or modification to optimize implementation in their context.

HIV-uninfected individuals who discontinue PrEP: To gain a deep understanding of the factors and the extent to which they influence willingness of HIV uninfected women to use PrEP regularly, we will conduct in-depth interviews and brief surveys with up to 300 women who initiate but later discontinue PrEP. We will probe for women's decision-making process related to HIV prevention methods use and alignment of perceived HIV risk. In addition, we will probe health systems related questions – waiting times, experiences at the clinic, and quality of services.

DATA

Data collection: All patient medical records will be captured through standard clinic data collection tools: FP registers; risk assessment screening tool and PrEP clinical encounter form (a clinical tool developed by NASCOP to deliver PrEP in clinical settings), MoH PrEP M & E tools, and tools developed for partner testing. As detailed, in colloboration with NASCOP and FP program some existing tools may be adapted to suite the FP context (e.g addition of MOH HIV prevention and PrEP indicators into FP register). As part of progrom evaluation, we will use multiple data sources (**Table 6**) to understand delivery and robustly capture, synthesize, and triangulate PrEP delivery processes to identify contextually relevant strategies for successful implementation, as well as practical difficulties in adoption, integration, and maintenance of integrated implementation of HIV prevention and care with FP services to inform wider implementation. Data sources will be from both program activities and as well as the research component of the project, including: 1) Qualitative interviews (policy key informants, providers, clients); 2) Quantitative questionnaires (providers, clients), and 3) TA visit summaries and observation reports. We will use the Consolidated Framework for Implementation Science Research (CFIR)[49] and other relevant theories and /or frameworks to guide development of data collection instruments, data analysis, and reporting of findings. We will investigate relevant constructs within the 5 domains CFIR: intervention characteristics (e.g. adaptability, cost), outer setting (e.g., MOH and county policies and resources), inner setting (e.g., structural characteristics of the clinic, training needs, clinic culture and implementation climate, relative priority, clinic goals and feedback leadership engagement, available resources), characteristics of individuals (e.g., staff self-efficacy to deliver PrEP), and process (e.g., planning, engaging, executing, and reflecting, emergence of champions). All data will be entered into passward-protected tablets daily and uploaded to web-based encrytped Rearch Electronic Data Capture (REDCap) servers. Program de-identified data that may be abstracted may include records on: demographics; behavior-risk characteristics, HIV testing, partner self-testing, PrEP uptake, adherence to PrEP, adverse events, FP use. Internal quality control reports will be run on a monthly basis to monitor program progress, discussed with clinics, MOH/ NASCOP and Kisumu County to improve implementation as the program comes to full scale.

Table 6: Data Sources

Data source	Description	Purpose
**Data abstraction**	▪ Data abstracted from clinical delivery tools	▪ Define HIV risk of women, PrEP initiation and continuation
**Technical assistance**	▪ TA reports prepared at baseline and 6-monthly	▪ Document detailed knowledge of process of adoption and integration of PrEP delivery and track changes in PrEP implementation processes.<br>▪ Rapid cycle analysis for quality improvement
**Qualitative interviews: user, provider, and key informants**	▪ Purposefully sampled patient, provider and key informants involved in the delivery	▪ Gain deep understanding of process of adoption and integration of PrEP delivery and track changes in PrEP implementation processes.
**Time & motions studies**	▪ Primary data collection	▪ Economic evaluation
**Exit interviews**	▪ Random on spot user structured surveys at the end of clinic visit	▪ User experiences and satisfaction
**Random urine and blood draw**	▪ Urine, plasma and DBS ~20% visits from PrEP users	▪ Objective assessment of adherence (tenofovir levels).
**Quantitative surveys, checklist**	▪ ORIC tool and questionnaire with provider and clinic mangers involved in the deliver	▪ Understand both individual level and program level outcomes, as well as process of adoption, integration, fidelity of PrEP delivery
**Patient actors**	▪ Unannounced visits to clinics by trained patient actors to complete standardized checklist on what services offered and quality of services	▪ Identify weak points in our FP-based PrEP delivery care pathway for women and scenarios that are of particular interest
**Observation**	▪ Informal	▪ Track changes in PrEP implementation processes.

Delivery and implementation outcomes: Evaluation of implementation outcomes will be framed by the RE-AIM and CFIR (as detailed in the preceding paragraph) frameworks. The RE-AIM guides evaluation of the public health impact of complex interventions, capturing formative, process, and outcome dimensions on the individual, organizational, and policy levels and gives a multi-dimensional understanding of the core components that drive public health impact. Reach, will be defined as proportion and subgroups of women accessing FP services who are screened and take PrEP, Effectiveness, will be assessed at individual level as 1) PrEP adherence, quantified by tenofovir levels in dried blood spots, and 2) women staying HIV free; Adoption will be assessed as the proportion of 1) clinics approached which implement intervention 2) targeted providers who are trained and implement PrEP thereafter; fidelity to PrEP delivery will be assessed as proportion of core components delivered per protocol; and Maintenance, a measure sustainability over time, will be the number of clinics delivering PrEP at 6 and 12 months after the intensive technical assistance .

Analysis for quantitative data: For each outcome, we will compare outcome during periods of usual care vs.

periods of intensive care in baseline and steps 1-3 to estimate a relative risk (RR) for the outcome associated with implementation. We will estimate each RR using a generalized mixed model. The key predictor will be intervention status of the clinic during the step in which the participant is enrolled, as a fixed effect. The model will also include step, as a fixed effect, and clinic, as a random effect. The model may also include a random effect for step and for the intervention effect, if evidence in our data shows that these are in fact varying randomly and not fixed. The mixed model will generate RRs using a modified Poisson model (i.e. Poisson regression with robust error variance); with robust standard errors to allow valid estimation of SEs for log (RR) from Poisson regression[54], which is a very stable model compared to alternatives. Using Steps 3-4 vs 7-8 (see **Figure 2**), we will conduct secondary analyses using similar methods to estimate changes in outcome during the maintenance phase compared to the intensive phase to quantitatively ascertain level of institutionalization. We will also describe, over the steps, the proportion of clients screened for HIV prevention including HIV testing and PrEP counseling, to assure that the intervention is being implemented as expected and ensure that any increase in % screening for HIV risk observed, includes PrEP counseling as part of the standard package now reaching a larger proportion of women. For individual level data from open-cohort, descriptive analyses will be done at baseline and over time, and appropriate regression models (e.g., cox proportional hazards for survival outcomes, and generalized estimating equation for binary or count data) comparing clinical outcomes between women who initiated PrEP vs those who chose not to initiate PrEP, accounting for age, partner HIV status, repeated measures, time-dependent exposures, and clinic-clustering.

*Analysis for qualitative data***.** Interviews will be recorded (after consent), transcribed, and translated into English (when necessary) by the study team. Transcripts will be reviewed separately by two investigators for completeness and initial theme generation. Interviews will be analyzed as they are collected and will use rapid debriefing reports to obtain real-time information and generate actionable points that will be feedback to clinics for possible adoption.[55] Coding and analysis will be performed in Atlas.ti or similar software, using a directed content analysis approach informed by constructs within the CFIR to summarize emerging themes and how they influence implementation. Coding results will be reviewed for consistency of text segmentation and code application, with continued evaluation of inter-coder agreement, and inconsistent results will be discussed by the coding team until consensus is reached. Queries will be used to abstract related themes, and themes will be grouped together into larger themes through group deliberative processes. We triangulate qualitative and quantitative data together to answer relevant questions about delivery process and outcomes simultaneously.

HEALTH ECOMOMICS STUDIES

We will conduct micro-costing studies during implementation of PrEP delivery in FP clinics and the current practice (baseline period) using activity-based approaches for costs incurred (start-up activities, clinic wide trainings, recruitment, PrEP service delivery, including HIV testing, PrEP drugs, STI treatment, creatinine testing, and follow-up) and direct program costs averted (health costs saved by averting HIV infections, personnel costs averted as result of providing FP and PrEP in one location). Program costs will also be collected from the study budget, public health clinic budgets, government reports, and the health economics literature, as we have done in previous work[56-59]. The primary outcome will be the costs (incremental relative to current practice) for PrEP provision in FP clinics including HIV treatment costs averted as a result of preventing HIV infections.

Time and motion studies: We will conduct time and motion studies by observing clinical visits to assess staff time spent on counseling, HIV counseling and PrEP provision. We will conduct staff interviews to assess time spent on PrEP-related activities and its impact on other usual FP services. We will serially measure total time spent to deliver PrEP integrated in FP clinics compared to staff time spent to deliver FP services in clinics during baseline period (with current practice) and during implementation period. We will assess costs incurred by patients (opportunity and financial costs) to access FP and HIV service including PrEP counseling and provision through participant questionnaires.

Decision analysis modeling: We will use a dynamic transmission model to simulate HIV infection, informed by previous modeling work[60, 61]. The model will explicitly capture current and future HIV prevention methods mix and the impact of integrating PrEP delivery in FP clinics on incident HIV infections as well as impact on current FP services. The model will be programmed in Excel (Matlab). We will estimate primary study outcomes: the cost of PrEP provision per person and incident HIV infection averted annually relative to current FP services alone. Models will be used to simulate the number of HIV infection cases in the absence of an intervention as well as current service (condom use, partner testing, treatment as prevention, STI screening and treatment) and

potential future HIV prevention methods mix. Total program costs will be divided by the number of HIV cases averted annually. We will additionally estimate the long-term costs and health effects that cannot be accurately assessed during our tracking period – using adapted models and data from the study and the literature.

Approach for estimating affordability and budget impact: Using the intervention effectiveness, costing, and decision analysis from this study, the cost per incident HIV case averted will be estimated. Using data on the incremental cost of the intervention, we will assess the budget impact on the Kenya MOH and Kisumu County budgets for including the interventions, incorporating the eligible population size, and current MOH and Kisumu budget expenditures. We will consider direct program costs, to ensure that measurements of MOH costs reflect the opportunity cost of the resources used in delivering services. The primary analysis will be from the *programmatic perspective* and secondary analysis from the *societal perspective* to account for users' financial and opportunity costs of accessing services. To the extent possible, we will use guidelines to facilitate standardization of cost data collection and reporting. This approach will also increase the transparency and generalizability of our results. For all key inputs and outputs, we will follow standard practices,[62] including the guidelines by the Panel of Cost-Effectiveness in Health and Medicine.[63] The primary analysis costs will be undiscounted inputs. Beyond scope of this grant, the unit costs estimated will provide the basis for more sophisticated cost-effectiveness analyses of the approaches in Kenya, for policy decision makers and payers.

QUALITY ASSURANCE PROCEDURES

All program procedures will be implemented in accordance with the MoH protocols and guidelines. All data will be maintained in a secure location. Internal quality control reports will be run on a monthly basis to monitor program progress, discussed with clinics, MOH/ NASCOP and County Health leadership to improve implementation as the program comes to full scale.

DISSEMINATION

We will disseminate outcomes of M & E activities to national and international stakeholders and facilitate technical assistance for PrEP scale-up in other settings. Our finalized PrEP delivery model for women will be collated in stakeholder consultations and training programs coordinated by NASCOP. The delivery outcomes will include identification of efficient and cost-effective model of delivering PrEP in FP clinics, operationalization and adaptation of MoH/NASCOP PrEP delivery tools in FP clinics, counselling messages, flow charts/posters tailored for women in FP clinics and providers to work through prevention options, staffing requirements, and summaries of key scientific aspects learned during the implementation period. We will work with the national program in delivery of PrEP to optimize supply chain management of PrEP drugs, development and dissemination of provider guidelines and PrEP initiation/monitoring checklists, counselling guidelines, and clinician technical support. We will convene a national stakeholder meeting, including other parts of government and prevention providers, civil society, academia, and others to set the stage for next steps for full-scale implementation in FP clinics.

HUMAN SUBJECTS CONSIDERATIONS

All program activities will be implemented through the standard routine FP clinic process. Screening of women for risk of acquiring HIV and provision of PrEP for those eligible and interested will be conducted as part of regular care in the PrEP program as stipulated by the Kenyan National Guidelines. Oral consenting will be implemented for all program activities as is the case for all standard of care services. Any individual who participates in the research component of the project, including provision of urine and blood samples to test for presence of tenofovir levels in the body, qualitative and quantitative surveys will provide an informed written consent. The consent forms will be translated into local languages.

The protocol, implementation plan, informed consent forms, data collection tools, and patient education materials will be reviewed and approved by the University of Washington Human Subjects Review Committee and the Kenyatta National Hospital-University of Nairobi Ethical Review Committee. Subsequent to initial review and approval, the responsible IRBs/ECs will review the program at least annually.

Informed consent

Informed consent will be conducted in a tiered fashion: oral consenting for routine program activities that directly impact patient care (all procedure key for provision of PrEP as standard of care, HIV testing and PrEP provision) and conducted as part of standard services. As is the case for all routine services, oral consenting for routine services will be done by the clinic providers. For all research procedures (i.e., urine, plasma and DBS samples

for drug levels, quantitative surveys, and qualitative interviews, phone/SMS support for adherence to PrEP) that are not directly routinely used for patient care but are important for overall program success and evaluation, written informed consent will be obtained before any procedure. Forms will be translated into local languages and verified by performing an independent back-translation.

Oral consenting for program activities

Clinic delivery staff will perform oral consenting for all standard consent information describing programmatic rollout and standard procedures for Screening for HIV risk, PrEP counseling and delivery (i.e. HIV testing, promotion of male partner HIV testing, STI screening and treatment, PrEP prescription, program data abstraction, PrEP adherence support and clinic follow up). Consenting will cover any additional questions or concerns participants may have. All efforts will be made to ensure that women have a thorough understanding of the program and that their decision to be in the program is optional, and that not participating will not in any way affect their usual care. Every effort will be made to protect patient privacy and confidentiality to the extent possible and only authorized person may have access to program records.

Justification for oral consenting for program activities

The goal of the program is to integrate PrEP delivery for HIV prevention as part of routine services offered to women receiving care in FP clinics in Kenya. Conducting oral consenting for non-research procedures in this program will be consistent with standard procedures implemented in providing routine services in the busy MCH and FP clinics in Kenya including provision of PMTCT, HIV testing, STI screening, immunization, and cervical cancer screening. The proposed program procedures will be part of routine HIV prevention services offered to women attending FP clinics. The procedures will represent no more than minimal risk and involves procedures for which consent would not normally be obtained outside the research context. The waiver of written consent will in no way affect the rights and welfare of the women receiving these services. We anticipate that utilizing oral consent will result in limited disruption of routine FP services and will facilitate seamless integration of PrEP counseling and delivery in family planning clinics, which is a key intervention area for the Kenya Ministry of Health HIV Prevention Revolution Road Map to maximize efficiency in service delivery through integration.

Justification for non-assent for emancipated minors (15-17 year old women).

The proposed program is integrating PrEP counseling and delivery as part of routine services to women of reproductive age attending FP clinics, including women aged 15 to 17 years (emancipated minors) who receive care from these clinics. HIV screening and provision of PrEP for girls ≥15 years is sanctioned in Kenya national guidelines for PrEP. It is expected that women 15-17 years who are already receiving routine services from antenatal and family clinics will be emancipated minors. 'Emancipated minors' are legally able to consent themselves for program activities and medical research. We will take the necessary steps to confirm that adolescents who consent to participate in research activities are emancipated minors as per local Kenya guidelines, by using pre-screening questions that include their age, marital status, pregnancy history, source of financial support, and whether they reside with parents. All proposed program activities will be implemented according to national guidelines and current routine practices in FP clinics. The Kenyan National Guidelines for HIV Testing and Counselling references emancipated youth and adolescents and notes that "children may be tested with the consent of a parent or guardian, or may give their own if they are symptomatic, pregnant, married, a parent, or engaged in behavior that puts them at risk of contracting HIV,". Similarly, the 2015 HIV Testing Services (HST) Guidelines in Kenya state also reduced the age for HTS without the guardian/parent consent to 15 years including all emancipated minors who may be below 15 years of age and the guidelines define an emancipated minor as "A person who is not legally an adult but who, because he or she is married, a parent or otherwise no longer dependent on the parents." The National Reproductive Health Research Guidelines also details that "unless specific legal provisions exist, consent to participate in research should be given only by the adolescents".

Consenting for research procedures.

All research related activities (procedures not directly related to standard of care provision of PrEP (e.g., interviews, questionnaires, blood draw for drug levels), informed consent will be obtained before any procedures are conducted. Consenting will be conducted by the research team. <u>Written informed consent</u>: We will perform paper-based and/or electronic-based consenting of participants. For the electronic version, we will program the

complete approved consent forms with signature pages onto tablets and participants will read along on the tablet during the consenting process. After we have ensured that participants have read and understood the consent forms, we will ask them to append their signature or put mark on the tablet. The signature will be stored electronically. Women will be given a paper copy of the consent information to take home with them if they choose to have it. This process will greatly reduce the burden of storing paper-based consent forms at each of the public health clinics, an activity that would require dedicated safe storage space and staff time for filing. Standard paper-based consent process will be available for those who do not wish to use the tablet-based electronic consent version or in case of a breakdown of electronic system. Oral informed consent for phone/SMS support for adherence to PrEP, partner HIV testing outcomes, and women who discontinue PrEP**:** Given the programmatic nature of this work and in order to minimize barriers to access to these recommended HIV prevention services including reducing participant's clinic time, we will implement oral informed consent for phone/SMS to support adherence to PrEP and proactively obtaining partner HIV testing outcomes, and women who discontinue PrEP. Oral informed consenting process will proceed as follows: After women have been seen by the usual clinic staff for program services, women who express interest in partner HIV testing and/or those who initiate PrEP, will be invited to participate in follow-up with phone/SMS to obtain outcomes on partner testing and/or support for adherence for those who initiate PrEP. Women will then be seen by a program nurse who will conduct the oral informed consenting process. In additional women who initiate but later discontinue PrEP will be contacted via phone for possible participation in a brief survey or interview about their experiences with clinic services and reasons for stopping PrEP use. Oral informed consent will involve meaningful discussion of program procedures and purpose of this evaluation. Women will have an opportunity to ask questions during or after the oral informed consenting process. To ensure we are clearly describing to the subjects what their particular experience will be, we will highlight the parts of the oral informed consent guide for each concept (e.g. partner testing or adherence support) the program nurse will need to cover with each subject. We will make it clear to all women that phone/SMS support for partner testing and or PrEP adherence (if relevant) are the research components and are voluntary. If a woman wants to opt out of the research aspect of the program, they will still be provided usual services including PrEP for anyone as per national guidelines on PrEP regardless of women's involvement in this research component. Every effort will be made to protect patient privacy and confidentiality to the extent possible and only authorized persons will have access to program records. Participants in the qualitative interviews will be compensated for their time consistent with the responsible local IRBs/ECs guidelines.

Risks

For this PrEP implementation roll-out program, where the goal is real-world delivery of PrEP and not medication themselves, the risks are expected to be minimal and will be those expected in routine care. Participants may become embarrassed, worried, or anxious when talking about their personal history, their sexual history, ways to protect against infections passed during sex, and their test results. Women may become embarrassed, worried, anxious or may become worried or anxious while being counseled about HIV risk or waiting for their HIV test results. Women who test and learn that they are HIV-positive may experience anxiety or depression related to their test results. As part of standard care services, HIV counseling will be provided by clinic staff who will been trained in specific issues related to HIV screening and PrEP, including stigma, blame, methods to avoid transmission, and available support services.

Every effort will be made to protect participant privacy and confidentiality. However, it is possible that participants' use of PrEP or involvement in research procedure could become known to others, if they are seen taking pills or coming back regularly for PrEP refill. Women could be treated unfairly or discriminated against, or could have problems being accepted by their families and/or communities. However, we anticipate that integration of FP and HIV prevention together in one-location will mitigate these concerns and will be appealing to women.

Risks and side effects related to PrEP include: occurring in a minority of individuals taking PrEP - gastrointestinal intolerance, such as nausea, diarrhea or vomiting, flatulence typically during the first month after PrEP initiation; rare but serious side effects kidney dysfunction mostly for those , including cases of acute renal failure and Fanconi's syndrome (renal tubular injury with severe hypophosphatemia), increase in bone metabolism leading to osteopenia, and hypersensitivity reaction. The risks of side effects of antiretroviral treatment (ART) are similar to those of PrEP and also rare but serious complications such as lactic acidosis.

Protection against risk

Study procedures will include qualitative interviews and prospective observational follow-up, HIV testing, blood collection, assessment of uptake and adherence PrEP for HIV prevention. PrEP will be provided to HIV-negative women by the clinic delivery staff as part of standard of care service, which will follow Kenya national guidelines. Counseling about antiretroviral-based HIV-1 prevention will include messaging describing the benefits of all strategies, based on evolving available data and national policies / national roll-out of antiretrovirals, including the Kenya guidelines of ART for all HIV-infected persons and PrEP for HIV prevention. For data collection, standardized questionnaires will be used that will include delivery questions as well as questions on sensitive topics, including sexual behavior, partner HIV status, and stigma. We have extensive experience with these questionnaires from our prior studies and the expertise and counseling resources required to attend to study participants. We anticipate the risks from the anticipated activities will be no greater than those expected from clinical care or our previous studies for research purposes. As with our other studies, no identifying information will be transmitted to the Coordinating Center at the University of Washington. We consider the risks associated with the project are small and minimal. The benefits for this work are consistent with clinical care benefits and cultural expectations and they follow the established standard with IRB/ERC approval in our other studies. We therefore believe the balance of benefit and risk is appropriate.

Benefits

As standard of care service, HIV-negative women will benefit by having access to ongoing HIV counseling and PrEP for HIV prevention. There may be no other direct benefits to participants in this study. However, participants and others also may benefit in the future from information learned from this study. Clinic delivery staff will provide all women with HIV prevention services, including promotion of condoms, partner testing, STI treatment, and ongoing HIV support, including counseling and referral to other support services as per standard of care practices. HIV prevention practices, according to national guidelines, will be provided to all women enrolled in this study. There are also possible benefits from ongoing access to HIV risk reduction counseling and other prevention services at the delivery clinics sites. In addition, participants and others also may benefit in the future from information learned from this study. The outcome of the project will be evidence upon which to based policy guidelines for scaling up HIV prevention centers in Kenya and nearby countries with similar HIV prevention needs. The family planning clinics that serve as sites for this project will be models upon which future centers can be based. Summary outcomes from this project will be submitted to overseeing regulatory bodies and will be especially important for the development of best practices and policy guidance.

Pregnancy detected during the study

Women who become or may become pregnant will be counseled on the known risks of PrEP use in pregnancy. Available human and animal data suggest that tenofovir-based PrEP does not increase the risk of major birth defects overall compared to the background rate, although well-controlled data are limited. Current FDA labeling for tenofovir disoproxil fumarate/emtricitabine is permissive to the use of PrEP in pregnancy and the Kenya PrEP guidelines permit use of PrEP by pregnant women at substantial risk of HIV acquisition, as cardinal pillar for PMTCT. Women who become pregnant while taking PrEP, will be counseled by clinic staff as part of standard practice which includes discussion about potential risk and benefits, taking into account the potential increased risk of HIV infection during pregnancy.

Care for persons identified as HIV-positive

The project will identify persons who are HIV-positive, either as part of the screening process of women or during follow-up. Clinic delivery staff will provide participants with their HIV test results in the context of post-test counseling as standard practice in Kenya. Women identified as HIV-positive will be immediately linked to treatment at the same clinic or clinic of their choice.

Benefits to the community

An important goal of this study is to achieve the study objectives in a way that provides benefits to the community that endure beyond the proposed study lifetime regardless of the specific outcome of the study. Some of these community benefits are listed below:

<u>Development of best practices and strategies to reach at risk women and access to prevention care:</u> The project

will enhance the existing systems in FP clinics to encourage a full complement of combination HIV prevention services, including offer of PrEP. Procedures to provide men with HIV self-testing as an alternative to clinic-based HIV testing will be developed, including simple strategies for teaching how to use and interpret HIV self-tests. The results of the study, and self-testing procedures will be provided to the Kenya Ministry of Health for inclusion in national HIV testing guidelines.

<u>Strengthening of capacity for combination of HIV prevention in family planning clinics</u>: The capacity developed at the participating family planning clinics in collaboration with the local County Government through training of clinic delivery staff will be a beneficial resource to the community well after the study is completed. The family planning clinics that serve as sites for this project will be models upon which future centers can be based. Summary outcomes from this project will be submitted to overseeing regulatory bodies and will be especially important for the development of best practices and policy guidance.

Importance of the knowledge to be gained

Knowledge gained from the studies proposed in this application will include information about implementation of PrEP and other HIV prevention methods in family planning clinics and optimization of the prevention of primary HIV infection reproductive age as a core pillar of PMTCT, which may have substantial impact on the global burden of HIV and progress towards an AIDS-free generation.

Confidentiality

Every effort will be made to protect participant privacy and confidentiality to the extent possible. Personal identifying information will be retained at the local study site and not forwarded to the University of Washington Coordinating Center. We will establish a standard operating procedure for confidentiality protection that reflects the local study implementation plan and the input of study staff and community representatives to identify potential confidentiality issues and strategies to address them. In addition to local considerations, the protections described below will be implemented.

All study-related information will be stored securely at the study site. All participant information will be stored in areas with limited access. Data collection, administrative forms, laboratory specimens, and other reports will be identified only by a coded number to maintain participant confidentiality. All records that contain names or other personal identifiers, such as locator forms and informed consent forms, will be stored separately from study records identified by code number. All local databases will be secured with password-protected access systems. Forms, lists, logbooks, appointment books, and any other listings that link participant ID numbers to other identifying information will be stored in a separate, locked file in an area with limited access.

Study oversight

The project will be subject to oversight by an independent external data and safety monitoring board (DSMB) established to oversee project implementation, data quality, monitor implementation and clinical outcomes, and patient safety. The committee will consist of expert clinicians, statisticians, policymakers and stakeholders from the HIV and SRH space in Kenya settings. The committee will meet 6-monthly to advise on study progress and whether the work aligns with Kenya MOH policies, and to offer guidance about results from international studies of PrEP that may spur policy change in Kenya. The DSMB will review data on HIV testing and counseling, partner HIV status, FP service use, PrEP uptake, continue and adherence, adverse outcome and will provide recommendations to the project implementation team as part of six-monthly reviews. Reports from all reviews will be submitted to overseeing IRBs/ECs.

PROGRAM RECORDS

Data ownership

The proposed program is a collaborative effort between KNH, NASCOP, Kisumu County and UW. The aforementioned institutions will jointly share ownership of the data. Authorship on publications, conference presentations, abstracts and other materials generated from this program will reflect contribution to design, execution and analysis of the program data.

Data release/sharing policy

All data collected as part of this implementation program will be made available without cost with written request and agreement to the data sharing agreement after completion of primary analyses. The data sharing agreement will ensure commitments to:

- Using the data only for program evaluation purposes and without attempting to identify patients
- Securing the data using appropriate computer technology
- Destroying or returning the data after analyses are completed
- Restrictions on redistribution of the data to third parties
- Proper acknowledgement of the data resource.

LIMITATIONS

As a programmatic project, characterization of some outcomes will be limited; the design was intentional to optimize program/policy relevance. Quality improvement and program outcomes will be evaluated using abstracted program data which may be is prone to missingness. We will work with facilities to harmonize data management systems in FP clinics and we will regularly share clinic-level data that can be used to improve services in the clinics.

TIMELINE (Table 7)

Table 7: Data Sources

AIM	MILESTONE	Calendar Years	2020				2021				2022				2023				2024				2025	
		Quarter	Q1	Q2	Q3	Q4	Q1	Q2	Q3	Q4	Q1	Q2	Q3	Q4	Q1	Q2	Q3	Q4	Q1	Q2	Q3	Q4	Q1	Q2
**Funding**	Funding Announced			■																				
**Protocol Planning**	Protocol Development			■	■																			
	Community and Stakeholder Consultation			■	■	■																		
	IRB Submission				■	■																		
**Operational Planning**	Stakeholder consultation					■	■																	
	SOP/Tools Development					■	■																	
	Project Staff training						■																	
	Project initiation						■																	
	Needs/Readiness to implement assessment (administer ORIC)					■	■	■																
	Workflow mapping and analysis						■	■	■	■														
**Protocol Execution**	Step-wedged implementation						■	■	■	■														
	Project implementation & follow of women who initiate PrEP						■	■	■	■	■	■	■	■	■	■	■	■	■	■	■			
	Open cohort follow-up for clinical outcomes						■	■	■	■	■	■	■	■	■	■	■	■	■	■	■			
	Urine and blood sample collection for adherence assessment						■	■	■	■	■	■	■	■	■	■	■	■	■	■	■			
	Qualitative interviews (subset) and analysis						■	■	■	■	■	■	■	■	■	■	■	■	■					
	Quantitative survey & analysis						■	■	■	■	■	■	■	■	■	■	■	■	■					
	Cost data & budget impact analysis						■	■	■	■	■	■	■	■	■	■	■	■	■					
	Urine and blood sample (plasma and DBS) analysis												■	■	■	■	■	■	■	■	■	■	■	
	Compile full spectrum of results for the project																		■	■	■	■	■	
**Dissemination**	Ongoing stakeholder engagement for planning, execution, and next steps						■	■	■	■	■	■	■	■	■	■	■	■	■	■	■	■	■	■
	Writing and results dissemination																			■	■	■	■	■

EXPECTED APPLICATION OF RESULTS

At the end of this project, we will have delivered integration of comprehensive HIV prevention and PrEP provision for at-risk young women in public family planning clinics in Kenya. Our findings of how to effectively promote integration of HIV prevention and family planning services will inform national and county governments about the feasibility of rolling out this integrated service to other facilities. Qualitative interviews with health managers and health providers will shed light on the facilitators and barriers to HIV prevention and PrEP care implementation in public health facilities. Cost-effectiveness data will guide policy makers' decisions about allocation of resources towards anti-retroviral based HIV prevention interventions. Finally, we will develop and

refine operational tools, clinical delivery products and assessment tools that will support delivery of HIV prevention services in family planning clinics at scale and will ensure that delivery continues at the national level

BUDGET (TOTAL BUDGET PERIOD) APPENDICES/ATTACHMENTS

This project is funded by the NIH/NIMH. The project fund will be available between January 2020 and April 2025. No salary support is requested for mentors (co-investigators and collaborators) as their funding is provided by other projects/sources.

REFERENCES

1. UNAIDS. UNAIDS data 2018; 2018 [updated 2018; cited 2019 September 03]; Available from: https://www.unaids.org/en/resources/documents/2018/unaids-data-2018.

2. A. Harrison, C. J. Colvin, C. Kuo, A. Swartz and M. Lurie. Sustained High HIV Incidence in Young Women in Southern Africa: Social, Behavioral, and Structural Factors and Emerging Intervention Approaches. Curr HIV/AIDS Rep. 2015;12(2):207-15. PMCID: PMC4430426.

3. J. Kinuthia, A. L. Drake, D. Matemo, B. A. Richardson, C. Zeh, L. Osborn, J. Overbaugh, R. S. McClelland and G. John-Stewart. HIV acquisition during pregnancy and postpartum is associated with genital infections and partnership characteristics. AIDS. 2015;29(15):2025-33. PMCID: PMC4692052.

4. J. Kinuthia, J. N. Kiarie, C. Farquhar, B. Richardson, R. Nduati, D. Mbori-Ngacha and G. John-Stewart. Cofactors for HIV-1 incidence during pregnancy and postpartum period. Curr HIV Res. 2010;8(7):510-4. PMCID: PMC3372399.

5. K. Shannon, K. Leiter, N. Phaladze, Z. Hlanze, A. C. Tsai, M. Heisler, V. Iacopino and S. D. Weiser. Gender inequity norms are associated with increased male-perpetrated rape and sexual risks for HIV infection in Botswana and Swaziland. PLoS One. 2012;7(1):e28739. PMCID: PMC3256140.

6. O. Evidence for Contraceptive and H. I. V. O. T. Consortium. HIV incidence among women using intramuscular depot medroxyprogesterone acetate, a copper intrauterine device, or a levonorgestrel implant for contraception: a randomised, multicentre, open-label trial. Lancet. 2019;394(10195):303-13. PMCID: PMC6675739.

7. A. S. Fauci, R. R. Redfield, G. Sigounas, M. D. Weahkee and B. P. Giroir. Ending the HIV Epidemic: A Plan for the United States. JAMA. 2019;321(9):844-5.

8. M. S. Cohen, Y. Q. Chen, M. McCauley, T. Gamble, M. C. Hosseinipour, N. Kumarasamy, J. G. Hakim, J. Kumwenda, B. Grinsztejn, J. H. Pilotto, S. V. Godbole, S. Mehendale, S. Chariyalertsak, B. R. Santos, K. H. Mayer, I. F. Hoffman, S. H. Eshleman, E. Piwowar-Manning, L. Wang, J. Makhema, L. A. Mills, G. de Bruyn, I. Sanne, J. Eron, J. Gallant, D. Havlir, S. Swindells, H. Ribaudo, V. Elharrar, D. Burns, T. E. Taha, K. Nielsen-Saines, D. Celentano, M. Essex, T. R. Fleming and H. S. Team. Prevention of HIV-1 infection with early antiretroviral therapy. N Engl J Med. 2011;365(6):493-505. PMCID: PMC3200068.

9. R. W. Eisinger, C. W. Dieffenbach and A. S. Fauci. HIV Viral Load and Transmissibility of HIV Infection: Undetectable Equals Untransmittable. JAMA. 2019;321(5):451-2.

10. J. M. Baeten, D. Donnell, P. Ndase, N. R. Mugo, J. D. Campbell, J. Wangisi, J. W. Tappero, E. A. Bukusi, C. R. Cohen, E. Katabira, A. Ronald, E. Tumwesigye, E. Were, K. H. Fife, J. Kiarie, C. Farquhar, G. John-Stewart, A. Kakia, J. Odoyo, A. Mucunguzi, E. Nakku-Joloba, R. Twesigye, K. Ngure, C. Apaka, H. Tamooh, F. Gabona, A. Mujugira, D. Panteleeff, K. K. Thomas, L. Kidoguchi, M. Krows, J. Revall, S. Morrison, H. Haugen, M. Emmanuel-Ogier, L. Ondrejcek, R. W. Coombs, L. Frenkel, C. Hendrix, N. N. Bumpus, D. Bangsberg, J. E. Haberer, W. S. Stevens, J. R. Lingappa, C. Celum and Partners PrEP Study Team. Antiretroviral prophylaxis for HIV prevention in heterosexual men and women. N Engl J Med. 2012;367(5):399-410. PMCID: 3920826.

11. R. M. Grant, J. R. Lama, P. L. Anderson, V. McMahan, A. Y. Liu, L. Vargas, P. Goicochea, M. Casapia, J. V. Guanira-Carranza, M. E. Ramirez-Cardich, O. Montoya-Herrera, T. Fernandez, V. G. Veloso, S. P. Buchbinder, S. Chariyalertsak, M. Schechter, L. G. Bekker, K. H. Mayer, E. G. Kallas, K. R. Amico, K. Mulligan, L. R. Bushman, R. J. Hance, C. Ganoza, P. Defechereux, B. Postle, F. Wang, J. J. McConnell, J. H. Zheng, J. Lee, J. F. Rooney, H. S. Jaffe, A. I. Martinez, D. N. Burns and D. V. Glidden. Preexposure chemoprophylaxis for HIV prevention in men who have sex with men. N Engl J Med. 2010;363(27):2587-99.

12. K. Choopanya, M. Martin, P. Suntharasamai, U. Sangkum, P. A. Mock, M. Leethochawalit, S. Chiamwongpaet, P. Kitisin, P. Natrujirote, S. Kittimunkong, R. Chuachoowong, R. J. Gvetadze, J. M. McNicholl, L. A. Paxton, M. E. Curlin, C. W. Hendrix and S. Vanichseni. Antiretroviral prophylaxis for HIV infection in injecting drug users in Bangkok, Thailand (the Bangkok Tenofovir Study): a randomised, double-blind, placebo-controlled phase 3 trial. Lancet. 2013;381(9883):2083-90.

13. World Health Organization. Guideline on when to start antiretroviral therapy and on pre-exposure prophylaxis for HIV Geneva: WHO; 2015 [updated 2015; cited 2016 December 30]; 78]. Available from: http://apps.who.int/iris/bitstream/10665/186275/1/9789241509565_eng.pdf.

14. P. M. Mugo, M. Micheni, J. Shangala, M. H. Hussein, S. M. Graham, T. F. Rinke de Wit and E. J. Sanders. Uptake and acceptability of oral HIV self-testing among community pharmacy clients in Kenya: a feasibility study. PLoS One. 2017;12(1):e0170868.

15. S. Masyuko, I. Mukui, O. Njathi, M. Kimani, P. Oluoch, J. Wamicwe, J. Mutegi, S. Njogo, M. Anyona, P. Muchiri, L. Maikweki, H. Musyoki, P. Bahati, J. Kyongo, T. Marwa, E. Irungu, M. Kiragu, U. Kioko, J. Ogando, D.

Were, K. Bartilol, M. Sirengo, N. Mugo, J. M. Baeten, P. Cherutich and E. P. T. W. G. O. Pr. Pre-exposure prophylaxis rollout in a national public sector program: the Kenyan case study. Sex Health. 2018;15(6):578-86.
16. United States Food and Drug Administration. Prescribing information for Truvada; 2012 [updated 2012; cited]; Available from: http://www.accessdata.fda.gov/drugsatfda_docs/label/2012/021752s030lbl.pdf.
17. World Health Organization. Guideline on when to start antiretroviral therapy and on pre-exposure prophylaxis for HIV. Switzerland; 2015 September 2015.
18. United States President's Emergency Plan for AIDS Relief- press release. 2014 [updated 2014; cited]; Available from: http://www.pepfar.gov/press/releases/2014/234531.htm.
19. US Public Health Service. Preexposure Prophylaxis for the Prevention of HIV infection in the United States. In: D. o. H. a. H. S. Centers for Disease Control and Prevention, editor.2014.
20. United Nations. The Millennium Development Goals Report; 2010 [updated 2010; cited 2019 September 03]; Available from: http://mdgs.un.org/unsd/mdg/Resources/Static/Products/Progress2010/MDG_Report_2010_En.pdf.
21. T. Powell-Jackson, J. Borghi, D. H. Mueller, E. Patouillard and A. Mills. Countdown to 2015: tracking donor assistance to maternal, newborn, and child health. The Lancet. 2006;368(9541):1077-87.
22. Kenya Ministry of Health. Demographic andHealth Survey; 2014 [updated 2014; cited 2019 August 30]; Available from: https://dhsprogram.com/pubs/pdf/fr308/fr308.pdf.
23. K. K. Mugwanya, J. Pintye, J. Kinuthia, F. Abuna, H. Lagat, E. R. Begnel, J. C. Dettinger, G. John-Stewart, J. M. Baeten, E. P. I. f. Y. W. Pr and P. Adolescents. Integrating preexposure prophylaxis delivery in routine family planning clinics: A feasibility programmatic evaluation in Kenya. PLoS Med. 2019;16(9):e1002885.
24. PAI. Integration means offering family planning/ reproductive health and HIV services; 2012 [updated 2012; cited 2019 September 03]; Available from: https://www.populationaction.org/data-and-maps/in-one-place-infographic/
25. R. Simmons, P. Hall, J. Diaz, M. Diaz, P. Fajans and J. Satia. The strategic approach to contraceptive introduction. Stud Fam Plann. 1997;28(2):79-94.
26. J. D. Auerbach, S. Kinsky, G. Brown and V. Charles. Knowledge, attitudes, and likelihood of pre-exposure prophylaxis (PrEP) use among US women at risk of acquiring HIV. AIDS Patient Care STDS. 2015;29(2):102-10. PMCID: PMC4321978.
27. S. Delany-Moretlwe, S. Mullick, R. Eakle and H. Rees. Planning for HIV preexposure prophylaxis introduction: lessons learned from contraception. Curr Opin HIV AIDS. 2016;11(1):87-93.
28. J. E. Myers, T. M. Ellman and C. Westhoff. Injectable agents for pre-exposure prophylaxis: lessons learned from contraception to inform HIV prevention. Curr Opin HIV AIDS. 2015;10(4):271-7.
29. Kenya Ministry of Health. Kenya HIV prevention revolution road map: Count down to 2030; 2014 [updated 2014; cited 2017 December 12]; Available from: http://www.nacc.or.ke/images/documents/Final.pdf.
30. L. Dwyer-Lindgren, M. A. Cork, A. Sligar, K. M. Steuben, K. F. Wilson, N. R. Provost, B. K. Mayala, J. D. VanderHeide, M. L. Collison, J. B. Hall, M. H. Biehl, A. Carter, T. Frank, D. Douwes-Schultz, R. Burstein, D. C. Casey, A. Deshpande, L. Earl, C. El Bcheraoui, T. H. Farag, N. J. Henry, D. Kinyoki, L. B. Marczak, M. R. Nixon, A. Osgood-Zimmerman, D. Pigott, R. C. Reiner, Jr., J. M. Ross, L. E. Schaeffer, D. L. Smith, N. Davis Weaver, K. E. Wiens, J. W. Eaton, J. E. Justman, A. Opio, B. Sartorius, F. Tanser, N. Wabiri, P. Piot, C. J. L. Murray and S. I. Hay. Mapping HIV prevalence in sub-Saharan Africa between 2000 and 2017. Nature. 2019;570(7760):189-93. PMCID: PMC6601349.
31. Kenya Ministry of Health. Framework for Implementation of Pre-Exposure Prophylaxis of HIV in Kenya; 2017 [updated 2017; cited]; Available from: Available at: http://www.prepwatch.org/wp-content/uploads/2017/05/Kenya_PrEP_Implementation_Framework.pdf. Accessed August 03, 2017.
32. R. E. Glasgow, T. M. Vogt and S. M. Boles. Evaluating the public health impact of health promotion interventions: the RE-AIM framework. Am J Public Health. 1999;89(9):1322-7. PMCID: PMC1508772.
33. I. J. Birdthistle, J. Fenty, M. Collumbien, C. Warren, J. Kimani, C. Ndwiga and S. Mayhew. Integration of HIV and reproductive health services in public sector facilities: analysis of client flow data over time in Kenya. BMJ Global Health. 2018;3(5):e000867.
34. W. Liambila, I. Askew, J. Mwangi, R. Ayisi, J. Kibaru and S. Mullick. Feasibility and effectiveness of integrating provider-initiated testing and counselling within family planning services in Kenya. AIDS. 2009;23:S115-S21.
35. J. P. Hughes, T. S. Granston and P. J. Heagerty. Current issues in the design and analysis of stepped wedge trials. Contemp Clin Trials. 2015;45(Pt A):55-60. PMCID: PMC4639463.

36. B. J. Weiner, H. Amick and S. Y. Lee. Conceptualization and measurement of organizational readiness for change: a review of the literature in health services research and other fields. Med Care Res Rev. 2008;65(4):379-436.
37. B. J. Weiner, M. A. Lewis and L. A. Linnan. Using organization theory to understand the determinants of effective implementation of worksite health promotion programs. Health Educ Res. 2009;24(2):292-305.
38. J. Cassidy. System analyzes readiness for integrated delivery. Health Prog. 1994;75(3):18-20, 2.
39. E. J. O'Connor and C. M. Fiol. Creating readiness and involvement. Physician Exec. 2006;32(1):72-4.
40. Y. T. Sweeney and C. Whitaker. Successful change: renaissance without revolution. Semin Nurse Manag. 1994;2(4):196-202.
41. B. J. Weiner. A theory of organizational readiness for change. Implementation Science. 2009;4(1):67.
42. C. M. Shea, S. R. Jacobs, D. A. Esserman, K. Bruce and B. J. Weiner. Organizational readiness for implementing change: a psychometric assessment of a new measure. Implementation Science. 2014;9(1):7.
43. Kenya Ministry of Health. Guidelines on the use of antiretroviral drugs for treating and preventing HIV infection in Kenya; 2016 [updated 2016; cited 2017 December 12]; Available from: http://emtct-iatt.org/wp-content/uploads/2016/09/Guidelines-on-Use-of-Antiretroviral-Drugs-for-Treating-and-Preventing-HI....pdf.
44. P. L. Anderson, D. V. Glidden, A. Liu, S. Buchbinder, J. R. Lama, J. V. Guanira, V. McMahan, L. R. Bushman, M. Casapia, O. Montoya-Herrera, V. G. Veloso, K. H. Mayer, S. Chariyalertsak, M. Schechter, L. G. Bekker, E. G. Kallas, R. M. Grant and T. iPrEx Study. Emtricitabine-tenofovir concentrations and pre-exposure prophylaxis efficacy in men who have sex with men. Sci Transl Med. 2012;4(151):151ra25. PMCID: PMC3721979.
45. D. Donnell, J. M. Baeten, N. N. Bumpus, J. Brantley, D. R. Bangsberg, J. E. Haberer, A. Mujugira, N. Mugo, P. Ndase, C. Hendrix and C. Celum. HIV protective efficacy and correlates of tenofovir blood concentrations in a clinical trial of PrEP for HIV prevention. J Acquir Immune Defic Syndr. 2014;66(3):340-8. PMCID: PMC4059553.
46. H. F. Hsieh and S. E. Shannon. Three approaches to qualitative content analysis. Qual Health Res. 2005;15(9):1277-88.
47. R. Grol and J. Grimshaw. From best evidence to best practice: effective implementation of change in patients' care. Lancet. 2003;362(9391):1225-30.
48. A. K. Rowe, D. de Savigny, C. F. Lanata and C. G. Victora. How can we achieve and maintain high-quality performance of health workers in low-resource settings? Lancet. 2005;366(9490):1026-35.
49. L. J. Damschroder, D. C. Aron, R. E. Keith, S. R. Kirsh, J. A. Alexander and J. C. Lowery. Fostering implementation of health services research findings into practice: a consolidated framework for advancing implementation science. Implementation Science. 2009;4(1):50.
50. K. Beima-Sofie, G. John-Stewart, B. Shah, D. Wamalwa, E. Maleche-Obimbo and M. Kelley. Using health provider insights to inform pediatric HIV disclosure: a qualitative study and practice framework from Kenya. AIDS Patient Care STDS. 2014;28(10):555-64. PMCID: PMC4183914.
51. K. M. Beima-Sofie, S. B. Trinidad, K. Ngure, R. Heffron, J. M. Baeten, G. C. John-Stewart and M. Kelley. Lessons from PrEP: A Qualitative Study Investigating How Clinical and Policy Experts Weigh Ethics and Evidence When Evaluating Preventive Medications for Use in Pregnant and Breastfeeding Women. AIDS Behav. 2019;23(7):1858-70. PMCID: PMC6570782.
52. J. Fairbanks, K. Beima-Sofie, P. Akinyi, D. Matemo, J. A. Unger, J. Kinuthia, G. O'Malley, A. L. Drake, G. John-Stewart and K. Ronen. You Will Know That Despite Being HIV Positive You Are Not Alone: Qualitative Study to Inform Content of a Text Messaging Intervention to Improve Prevention of Mother-to-Child HIV Transmission. JMIR Mhealth Uhealth. 2018;6(7):e10671. PMCID: PMC6072973.
53. J. M. Simoni, K. Beima-Sofie, K. R. Amico, S. G. Hosek, M. O. Johnson and B. S. Mensch. Debrief Reports to Expedite the Impact of Qualitative Research: Do They Accurately Capture Data from In-depth Interviews? AIDS Behav. 2019;23(8):2185-9. PMCID: PMC6642848.
54. G. Zou. A modified poisson regression approach to prospective studies with binary data. Am J Epidemiol. 2004;159(7):702-6.
55. J. M. Simoni, K. Beima-Sofie, K. R. Amico, S. G. Hosek, M. O. Johnson and B. S. Mensch. Debrief Reports to Expedite the Impact of Qualitative Research: Do They Accurately Capture Data from In-depth Interviews? AIDS Behav. 2019.
56. I. Golovaty, M. Sharma, A. Van Heerden, H. van Rooyen, J. M. Baeten, C. Celum and R. V. Barnabas. Cost of Integrating Noncommunicable Disease Screening Into Home-Based HIV Testing and Counseling in South Africa. J Acquir Immune Defic Syndr. 2018;78(5):522-6. PMCID: PMC6037552.

57. E. M. Irungu, M. Sharma, C. Maronga, N. Mugo, K. Ngure, C. Celum, R. V. Barnabas, J. Baeten and R. Heffron. The Incremental Cost of Delivering PrEP as a Bridge to ART for HIV Serodiscordant Couples in Public HIV Care Clinics in Kenya. AIDS Res Treat. 2019;2019:4170615. PMCID: PMC6521338.
58. A. N. Phillips, V. Cambiano, F. Nakagawa, L. Bansi-Matharu, D. Wilson, I. Jani, T. Apollo, M. Sculpher, T. Hallett, C. Kerr, J. J. van Oosterhout, J. W. Eaton, J. Estill, B. Williams, N. Doi, F. Cowan, O. Keiser, D. Ford, K. Hatzold, R. Barnabas, H. Ayles, G. Meyer-Rath, L. Nelson, C. Johnson, R. Baggaley, A. Fakoya, A. Jahn and P. Revill. Cost-per-diagnosis as a metric for monitoring cost-effectiveness of HIV testing programmes in low-income settings in southern Africa: health economic and modelling analysis. J Int AIDS Soc. 2019;22(7):e25325. PMCID: PMC6615491.
59. D. A. Roberts, R. V. Barnabas, F. Abuna, H. Lagat, J. Kinuthia, J. Pintye, A. F. Bochner, S. Forsythe, G. B. Gomez, J. M. Baeten, G. John-Stewart and C. Levin. The role of costing in the introduction and scale-up of HIV pre-exposure prophylaxis: evidence from integrating PrEP into routine maternal and child health and family planning clinics in western Kenya. J Int AIDS Soc. 2019;22 Suppl 4:e25296. PMCID: PMC6643078.
60. R. Ying, M. Sharma, C. Celum, J. M. Baeten, H. van Rooyen, J. P. Hughes, G. Garnett and R. V. Barnabas. Home testing and counselling to reduce HIV incidence in a generalised epidemic setting: a mathematical modelling analysis. Lancet HIV. 2016;3(6):e275-82. PMCID: PMC4927306.
61. R. Ying, M. Sharma, R. Heffron, C. L. Celum, J. M. Baeten, E. Katabira, N. Bulya and R. V. Barnabas. Cost-effectiveness of pre-exposure prophylaxis targeted to high-risk serodiscordant couples as a bridge to sustained ART use in Kampala, Uganda. J Int AIDS Soc. 2015;18(4 Suppl 3):20013. PMCID: PMC4509901.
62. M. Drummond and M. Drummond. Methods for the economic evaluation of health care programmes. 3rd ed. Oxford ; New York: Oxford University Press; 2005.
63. M. R. Gold. Cost-effectiveness in health and medicine. New York: Oxford University Press; 1996.
