# Supplementary material for: Oral preexposure prophylaxis use and the risk of bacterial sexually transmitted infections and HIV among African women: A prospective observational cohort study
Source: PLoS Med. 2026 Mar 9;23(3):e1004962. doi: 10.1371/journal.pmed.1004962 (PMC13002101; doi:10.1371/journal.pmed.1004962)
Supplement: S2 File — (PDF) [file pmed.1004962.s006.pdf]

The Family Planning plus HIV prevention (FP-Plus) Project

Statistical Analysis Plan for the Association of Oral PrEP use with STI diagnosis

Version 1.1

16 January 2024

Based on protocol version 1.0 dated 27 October 2020

Kenneth K. Mugwanya

David Mukasa

Contents

Contents 2
1. Introduction 3
2. Study Population 3
3. Study Design 3
3.1. Exposure of interest 4
4. Study Outcomes 4
5. Follow-up and Data collection 4
5.1. Sample size and power .... **Error! Bookmark not defined.**
6. General Statistical analysis Considerations 5
6.1. Missing values 5
7. Analysis 5
7.1.1. Descriptive statistics 6
7.1.2. Primary analysis 6
7.1.3. Secondary analysis 6
7.1.4. Baseline covariates 6
7.1.5. Pre-planned subgroup analyses 7
7.2. HIV seroconversion 7
8. Appendix 7

1. Introduction

This statistical analysis plan (SAP) describes the statistical procedures and analyses and data displays that address study objectives specified in protocol version 1.0 of the PrEP-aring family planning clinics to streamline integration of HIV prevention services for young women in Kenya study.

New versions of the SAP will be issued to document updates and changes in the plan. Any meaningful changes or additions to this SAP (e.g., in response to protocol amendments or violations of assumptions underlying pre-planned analyses), and the timing of such changes in relation to timing of performing the analysis will be documented in an Appendix of the SAP.

2. Study Population

Heterosexual sexually active HIV-negative adolescent girls and young women will be identified and have data abstracted from twelve participating family planning clinics in Kisumu County, Kenya.

Inclusion criteria

- Of reproductive age, >= 15 years
- Sexually active female
- Accessing services at clinics participating in PrEP program in the SWCRT
- Able and willing to provide consent for follow-up in the cohort
- HIV uninfected at baseline based on negative HIV tests, per Kenya national guidelines
- Has at least one risk factor which puts them at substantial on-going risk for HIV as defined in the in the Kenya PrEP national guidelines
- Enrolled in the study during the intervention period of the SWCRT

3. Study Design

The parent study is a stepped-wedge cluster-randomized trials (SWCRTs), incorporating a staged implementation of the HIV prevention intervention into 12 family planning clinics in Kisumu County, Kenya in the first SWCRT, followed by staged removal of the intervention in the second SWCRT.

In addition, a longitudinal open cohort study will be conducted within the SWCRTs, in which participants at five clinics will be engaged to join a cohort and be followed with research procedures to capture complementary outcome data. This cohort will only enroll participants during intervention and sustainment steps.

During the steps in which the intervention has already rolled out, a subset of high volume sites will continuously enroll women into a cohort and follow them quarterly.

4. Exposure of interest

The expsore of interest will be PrEP use among individuals to be eligible for PrEP based on the Ministry of Health PrEP guidelines. This stufy population includes both women who accepted and those who declined PrEP.

- PrEP use (exposure) will be ascertained through self-reported quantitative questionnaires on PrEP use and validated with clinic visit attendance and PrEP pharmacy refill records.
- PrEP exposure status will be categorized in two ways to better triangulate PrEP exposure classification throughout the 12-month period.
  - Binary variable based on PrEP initiation status ascertained at enrollment (i.e., initiated or not) as the primary exposure of interest.
  - Consistency of PrEP use during the first 6 months since enrollment with three categories: never used PrEP at all, inconsistently on PrEP, and consistently on PrEP. This secondary classification of PrEP use is justified given that PrEP use is dynamic, and users may cycle on and off PrEP, and those who initiate may go off PrEP just after initiation, conditioned on baseline PrEP initiation status.

5. Study Outcomes

Several individual-level outcomes will be measured and characterized in the cohort participants:

- Etiological STI diagnoses of *Chlamydia trachomatis* or *Neisseria gonorrhoeae* in urine ascertained at baseline and longitudinally.
  - Testing at baseline and batched testing of archived urine for follow up visits.
- Knowledge of partner HIV status
- PrEP adherence among those who initiate PrEP based on TFV-DP in DBS samples collected at 1 month after PrEP initiation and quarterly thereafter
- HIV diagnosis based on quarterly testing
- Type of contraception provided/received at baseline and longitudinally
- Sexual behavior at baseline and longitudinally

6. Follow-up and Data collection

- Cohort participants had had research visits at enrollment and 1, 3, 6, 9, and 12 months, with HIV testing at each visit.
- HIV testing was performed in line with the Kenya national HIV testing algorithms, using Determine HIV-1/2 and Fast Response test kits.
- At each scheduled visit, structured questionnaires were administered to obtain demographics, PrEP use status, HIV risk, contraception method, and HIV prevention methods use.

- Urine samples were tested for Neisseria gonorrhoeae and Chlamydia trachomatis using the GeneXpert CT/NG real-time PCR assay (Cepheid, CA, USA) for all participants at baseline and for a randomly selected subset at 3, 6, and 12 months due to logistical constraints.
- PrEP use will be ascertained through self-report (structured CRF) and pharmacy refill information from PrEP card.
- A subset of enrollees in the SWCRT who enroll after intervention rollout, have initiated PrEP at first (enrollment) visit, and consent to a blood draw for objective assessment of PrEP adherence. For budgetary and logistically and constraints, only a subset of these samples will be batched tested for TFV-DP levels at the end of the parent project.
- For the SWCRT, study data are entered into REDCap study database by the study data abstractors.
- For Cohort, electronic CRFs are administered in REDCap by research staff.

7. Sample size

Sample size for the longitudinal cohort determined based on feasibility of recruiting into the cohort. Planned to enroll up to 900 women total in the longitudinal cohort during the intervention phase of the SWCRT; rather than setting a goal of a certain number of women per clinic-step, the participants will be enrolled consecutively as quickly as possible.

Note: Sample size was adjusted to 650 one year after enrollement started, but before the end of the study.

8. General Statistical analysis Considerations

Data listings and summaries similar to those described here may be provided to the Data Safety and Monitoring Board (DSMB), but statistical testing or comparisons by arm will not be included. Analyses described in this plan are final analyses to be performed on the final dataset for the study.

8.1. Missing values

For the longitudinal cohort, individuals will be considered lost to follow-up if they do not have an HIV test within 12 months of enrollment in the cohort.

Variables with unreliable reporting will excluded from adjusted analyses, and indeterminate STI test results will be excluded from the analysis.

9. Analysis

- The primary hypothesis is that PrEP use is not associated with the risk of incident Chlamydia trachomatis and/or Neisseria gonorrhoeae diagnosis.
- The primary analysis will be analysis of the association PrEP use and etiological STI diagnosis. The analyses of the remaining outcomes (knowledge of partner HIV status, PrEP adherence, HIV

diagnosis, provision of contraception, type of contraception provided, and sexual behaviors) will be secondary and exploaratory.

- All analyses will use individual-level data collected during intervention implementation of the SWCRT.

9.1.1.Descriptive statistics

- We will provide tables and figures giving descriptive statistics of the outcomes overall and by exposure status.
- Continuous variables will be described with mean (SD) or median (Q1, Q3). Categorical variables will be described with N (%).

9.1.2.Primary analysis

- Modified Poisson generalized estimating equation (GEE) models with robust standard errors to estimate the relative risk (RR) with corresponding 95% confidence intervals (95% CIs) of infection with chlamydia or gonorrhoeae during follow-up by self-reported PrEP status.
- All enrolled participants, including those with a baseline STI diagnosis but only subsequent STI diagnoses (not baseline) will be considered as events in the primary analysis.
- Statistical tests will be 2-sided tests considered statistically significant if $p < 0.05$ (i.e., $\alpha=0.05$)
- ***Updated notes***: Due to budget constratints, the primary analysis models based on the binary baseline PrEP initiation exposure will use three STI outcome measurement time points (i.e., STI test results done 3, 6, and 12 months).
- For the exposure classification with three category PrEP use consistency 6 month since initiation, the analysis model will be based on two STI outcome measurement time points (i.e., STI test results at 6 and 12 months)

9.1.3.Secondary analysis

Exploratory secondary analyses will be conducted to provide contextual insight and inform appropraiate interpretation of primary analysis. These analyses will be exploratory hypothesis-generating and should be interpreted with caution. Secondary outcomes will include include:

- Pathogen-specific STI diagnoses (*Chlamydia trachomatis* and *Neisseria gonorrhoeae*)
- Longitudinally assessed sexual behavior measures (condomless sex at last sex, new sexual partners in the past 3 months, and transactional sex)
- HIV incidence overall and by PrEP use status.

9.1.4.Baseline covariates

- Covariates to be adjusted for in all models (as appropriate) determined apriori as potential confounders guided by the Directed Acyclic Graphs principles include age, STI diagnosis at enrollment, any contraceptive use, more than one sexual partner, education status, marital status, last partner HIV status, any transactional sex in 3 months pre-enrollment, and clinic site.

9.1.5.Pre-planned subgroup analyses

- Pre-planned subgroup analysis will be age categories and among those with STI diagnosis or treatment in 6 months pre-enrollment, STI at enrollment, and any contraceptive use at enrollment.

9.2. HIV seroconversion

- For the outcome of HIV diagnosis, we are interested in describing HIV incidence among those who do and do not initiate PrEP.
- Seroconversion will be determined by local HIV testing guidelines based on results of quarterly HIV tests in cohort participants. For initially HIV uninfected participants who seroconvert, a listing will be provided.
- For those who initiate PrEP, regardless of whether they adhere, the “clock” for HIV incidence will begin at the first quarterly visit after PrEP initiation.

10. Appendix : Documentation of updates and clarification

Date: 06/02/2025

- Due budgatry constratints, three STI outcome measurement time points (i.e., 3, 6, and 12 months) were included in the primary analysis models based on the binary baseline PrEP initiation exposure.
- Minor analytic refinements were made during manuscript revision in response to peer review and the rationale included refining covariate adjustment to strengthen epidemiologic validity, assess robustness through sensitivity analyses, and improve transparency regarding missing data, clustering, and analytic assumptions.
