# Supplementary material for: Oral preexposure prophylaxis use and the risk of bacterial sexually transmitted infections and HIV among African women: A prospective observational cohort study
Source: PLoS Med. 2026 Mar 9;23(3):e1004962. doi: 10.1371/journal.pmed.1004962 (PMC13002101; doi:10.1371/journal.pmed.1004962)
Supplement: S3 File — (DOCX) [file pmed.1004962.s007.docx]

**Suplementary Materials**

The FP Plus Project Team

University of Washington, Seattle, USA

Kenneth K. Mugwanya (Principal Investigator), Torin Schaafsma, Jennifer Morton (Project Director), Kristin M. Beima-Sofie, Bryan Weiner, PhD, Jared M. Baeten

**Research & Programs, Kenyatta National Hospital, Nairobi, Kenya**

John Kinuthia (Site Principal Investigator), Daniel Matemo (Project Coordinator), Cynthia Wandera, Esher Achieng, Irine Cherotich, Valarie Kemunto, Beatrice Oduor, David Owaga, Tecy Oyombra, Stephen Odhiambo, Mercy Bii

**Vaccine and Infectious Diseases Division, Fred Hutchinson Cancer Center, Seattle, USA**

Allison Meisner (Protocol Biostatistician)

**Collaborating Institutions**

University of Washington, Seattle, USA

Kenyatta National Hospital, Nairobi, Kenya

Kenya National AIDS/STD Control Programme, Nairobi, Kenya

Kisumu County Department of Health, Kisumu, Kenya

Fred Hutchinson Cancer Center, Seattle, USA

**Study data monitoring committee**

Dr. Frankline M. Onchiri (Chair)

Prof. James Kiarie

Dr. Kenneth Ngure

Prof. Elizabeth Bukusi

Prof. Kawango Agot (RIP)
